# Biofortification of dietary fibre: exploring enhanced β-glucan and arabinoxylan content in a panel of *Triticum* and wild relatives

**DOI:** 10.3389/fpls.2025.1660594

**Published:** 2026-01-22

**Authors:** Prexha Kapoor, Sourav Panigrahi, Yogita Singh, Sundip Kumar, Krishna Pal Singh, Farkhandah Jan, Reyazul Rouf Mir, Upendra Kumar

**Affiliations:** 1 Department of Molecular Biology and Biotechnology, College of Biotechnology, Chaudhary Charan Singh Haryana Agricultural University, Hisar, Haryana, India; 2 Stockbridge School of Agriculture, University of Massachusetts, Amherst, MA, United States; 3 Molecular Cytogenetics Laboratory, Department of Molecular Biology & Genetic Engineering, College of Basic Sciences & Humanities, GB Pant University of Agriculture & Technology, Pantnagar, India; 4 Biophysics Unit, College of Basic Sciences & Humanities, GB Pant University of Agriculture & Technology, Pantnagar, India; 5 Vice-Chancellor’s Secretariat, Mahatma Jyotiba Phule Rohilkhand University, Bareilly, India; 6 Division of Genetics and Plant Breeding, Sher-e-Kashmir University of Agricultural Sciences and Technology of Kashmir (SKUAST-Kashmir), Srinagar, India; 7 Department of Plant Science, Mahatma Jyotiba Phule Rohilkhand University, Bareilly, India

**Keywords:** wheat, β-Glucan, arabinoxylan, protein, starch, dietary fibre

## Abstract

Dietary fibres, especially non-starch polysaccharides including β-glucan and arabinoxylan from cereal grains, are vital for human health due to their role in lowering cholesterol, regulating glycaemic index, and reducing the risk of chronic diseases like type II diabetes. A daily intake containing 2% or more β-glucan is often associated with health benefits. Wheat (*Triticum aestivum* L.), a staple crop and major source of dietary carbohydrates, contains limited variability for these fibre components compared with its wild relatives. To explore genetic resources for fibre biofortification, we evaluated a panel of 478 wheat genotypes including 37 wild relatives, 6 tetraploid, and 435 hexaploid wheat accessions for β-glucan, arabinoxylan, alongside protein, and starch content. The panel showed wide variation, with mean values of 0.93% for β-glucan, 5.77% for arabinoxylan, 13.37% for protein, and 68.51% for starch. Among wild relatives, *Aegilops peregrina* and *Aegilops kotschyi* emerged as superior sources of high β-glucan and arabinoxylan, whereas modern cultivars generally exhibited lower values. Significant positive correlations were observed between β-glucan and protein, and negative associations with starch and thousand-grain weight, indicating potential trade-offs in grain composition. These findings highlight the untapped potential of wild genetic resources for enhancing the nutritional quality of wheat and provide promising candidates for pre-breeding and biofortification strategies aimed at improving dietary fibre in staple foods.

## 1 Introduction

Cereals are staple foods for sustenance and a crucial source of nutrition to humans. Their incorporation into various products has significant economic importance. In the world, wheat is the third most widely grown crop with a yearly gross yield of ~781.2 million tonnes in 2022 (FAO, 2022). Beyond its global production scale, wheat is distinguished from maize and rice by its diverse utilization in foods such as bread, noodles, and biscuits. It supplements ~20% of the everyday protein and energy, and it is a substantial source of many other nutrients that are good for human health, like dietary fibres (Lafiandra et al., 2014).

Despite its importance, majority of wheat grain is composed of starch (~60%–70% of the grain and ~70%–80% of the flour) and proteins (~10%–15%). However, the proportion of dietary fibre is relatively low compared with these major components, which limits wheat’s ability to meet recommended daily fibre intake. In contrast, dietary fibres, especially non-starch polysaccharides (NSPs) derived from the cell wall make up only approximately 3%–8% of the total constituents. This limited fibre content is concerning, as dietary fibre plays critical roles in human health.

Due to high adaptability, wheat consumption has significantly expanded in recent decades, which increases the importance of this cereal in human diets (Shewry and Hey, 2015) Numerous studies approved by the Food and Drug Administration (FDA) have shown that cereal grains dietary fibres are certainly connected with reduced cholesterol levels and lower glycaemic index (Lattimer and Haub, 2010; Nirmala Prasadi and Joye, 2020). A clinical investigation of the European Prospective Investigation into Cancer and Nutrition (EPIC) has also demonstrated a link between significance of whole grain consumption and a lower risk of metabolic syndrome, inflammatory bowel disease, colon cancer, and cardiovascular disease (Bingham et al., 2003; Crowe et al., 2011; Stephen et al., 2017). Health experts from all over the world recommend adults to consume 25–35 g of fibre daily; however, the average adult only consumes approximately half of this amount (Lunn and Buttriss, 2007; Stephen et al., 2017). The non-starch polysaccharides arabinoxylan and (1-3)(1-4)-β-D-glucan (β-glucan) are the main dietary fibre components in wheat grain, which make up around 70% and 20%, respectively, of the total cell wall polysaccharides in the starchy endosperm and consequently in white flour (Mares and Stone, 1973). The most significant of the two is β-glucan, which provides various health advantages including those related to faecal bulking, absorption of minerals, prebiotic effects, lower blood cholesterol, improved metabolic activities, and a decreased risk of type II diabetes (Collins et al., 2010). A concentration of 2% or higher β-glucan in wheat is considered sufficient to contribute to health benefits, such as reduction in blood cholesterol and glycaemic index (Wood, 2007; EFSA Panel on Dietetic Products Nutrition and Allergies, 2010; Marcotuli et al., 2019). Before being absorbed in the gastrointestinal tract, dietary digestible carbohydrates are hydrolysed to the monomeric sugars, glucose, or fructose making a gel-like formation which helps in easy digestion (Dona et al., 2010; Malunga et al., 2017).

To address this limitation, understanding the genetic diversity of wheat and its relatives is essential. Wheat exists in three major ploidy forms: diploid (2n = 14, AA), tetraploid (2n = 28, AABB), and hexaploid (2n = 42, AABBDD). Globally, hexaploid bread wheat (*T. aestivum*) constitutes ~95% of cultivation, whereas tetraploid durum wheat (*T. turgidum* ssp. *durum*) accounts for the remaining ~5% (Rakszegi et al., 2017; Marcotuli et al., 2020). Compared with other cereal crops like barley and oat, wheat has a relatively low level of dietary fibre content (Havrlentová and Kraic, 2006). To increase the amount of dietary fibre in wheat-based products, barley and oat flour have been added; nevertheless, performance of the flour, texture, and flavour are limitations when manufacturing baked goods, and there is not much economic adoption of such foods (Collins et al., 2010; Limberger-Bayer et al., 2014). Instead of relying on external supplementation, a more sustainable approach is to explore wild *Triticum* and *Aegilops* species, which harbour valuable genetic variation. These wild relatives possess a range of agronomically useful genes that can be transferred into cultivated wheat through intergeneric or interspecific crosses. Such approaches allow the development of chromosome introgression, addition, or substitution lines, thereby broadening the genetic base for dietary fibre improvement (Schneider et al., 2008; Giancaspro et al., 2016; Kumar et al., 2016; Rakszegi et al., 2017). Therefore, these can be a potential source of dietary fibre to fulfil the daily dietary requirements.

Nevertheless, dietary fibres significantly affect how wheat grain is utilized as animal feed, for milling and baking, because of their viscosity in various aqueous solution due to different hydration qualities. The amount of dietary fibres varies significantly between wheat cultivars and grain tissue (Saulnier et al., 2007; Pritchard et al., 2011). It has been suggested that genotype determined approximately 50% or more of the variation observed in wheat wholemeal for dietary fibre content, which is highly heritable (Gebruers et al., 2010; Shewry et al., 2013). In this scenario, the primary goal of the study was to examine a panel of wheat genotypes for the amount of dietary fibre they contained to find genotypes having high dietary fibre contents that might be used as an appropriate target for plant breeding and to advance economic interests.

## 2 Materials and methods

### 2.1 Seed material

A panel of 478 wheat genotypes was used for the estimation of dietary fibre components, including (1-3) (1-4)-β-D-glucan (β-glucan) and arabinoxylan, along with D-xylose, protein, starch, and thousand-grain weight. The panel comprised 37 wild relatives of wheat, 6 tetraploid, and 435 hexaploid genotypes, which are listed in **Supplementary Table S1** with their accession number, source, origin, and genotype group. Tetraploid and hexaploid wheat genotypes were selected from the germplasm collection available in the “Wheat and Barley section,” Department of Genetics and Plant Breeding, Chaudhary Charan Singh Haryana Agricultural University, Hisar, Haryana, India, and also procured from the Indian Institute of Wheat and Barley Research, Karnal, Haryana, India. Wild relatives of wheat were procured from ICAR-National Bureau of Plant Genetic Resources, New Delhi, India.

### 2.2 Experimental design and crop management

The entire panel of 478 wheat genotypes, comprising cultivated and wild relatives, were grown in the fields of the Research Area Wheat Section, Department of Genetics & Plant Breeding, Chaudhary Charan Singh Haryana Agricultural University, Hisar, Haryana, India (29° 8′ 58.5312″ N, 75° 41′ 39.0444″ E) in the *rabi* season of 2020–21 and 2021-22. The seeds were sown in a double row of 1 m in length with a 20-cm distance between each row and 80 cm between blocks in a randomized complete block design with three replications. All advised and recommended agronomic procedures were followed to grow the wheat crop. The commercial ZnSO_4_·7H_2_O, together with 50% of the advised 200 kg ha^−1^ of nitrogen and 100% of 50 kg ha^−1^ of phosphorus fertilizers, was used as the base fertilizer in the field. After 25 days of sowing of wheat, the remaining 50% or 100 kg ha^−1^ of nitrogen, was applied as top dressing in the soil. Whole plots were manually picked at maturity, and 10 g of grain samples from each plot was utilized for biochemical analysis. Thousand-grain weight (1,000 GW) was derived from the mean of three separate samples by counting and weighing 100 grains of each genotype and then multiplying the weight by 10.

### 2.3 Preprocessing of wheat grains

The samples of wheat grains were dried in a hot air oven at a controlled temperature of 60°C to a constant weight. Around 10 g of wheat grain from each genotype was ground into flour by a ball mill homogenizer (Domel, Millmix 20) at a frequency of 27 for 3 min to get a uniform flour and stored in airtight containers for further evaluations. For each genotype, grains were harvested from three biological field replicates and pooled, and a representative sample was analysed in duplicate in the laboratory.

### 2.4 Estimation of β-glucan in wheat grains

The concentration of β-glucan content was estimated in whole-grain wheat flour using a Mixed-Linkage β-glucan Assay Kit (Megazyme International Ireland Ltd, Wicklow, Ireland), based on the acclaimed method by McCleary and Codd (Mccleary and Codd, 1991). The streamlined procedure for the estimation of β-glucan was used for two replicates of each genotype as per the instructions and procedure prescribed by manufacturers following the AACC method 32–23.01, AOAC Method 995.16, and ICC Standard Method 166 including the barley β-glucan (4.1%) as reference standard.

### 2.5 Estimation of arabinoxylan and D-xylose in wheat grains

Arabinoxylan content was quantified using a scaled-down version of the Megazyme D-xylose Assay Kit (K-XYLOSE 04/17, Megazyme, Ireland). The D-xylose released during enzymatic hydrolysis was quantified spectrophotometrically, and the arabinoxylan content was calculated by multiplying the molecular weight conversion factor from the xylose monomer to the arabinoxylan polymer. Two laboratory replicates of each genotype were used for the analysis.

The D-follows:


$$\text{c}=\frac{\text{FV}\times \text{MW }}{\text{ϵ}\times \text{d}\times \text{v}}\times \text{ΔA }(\text{D}-\text{xylose})\;({\text{gL}}^{-1})$$

where:

FV = Final volume (mL).

MW = Molecular weight of D-xylose (g mol^−1^).

ϵ = Extinction coefficient of NADH (340 nm).

6300 (l × mol^−1^ × cm^−1^).

d = Light path length (cm).

v = Sample volume (mL).


$$\begin{array}{l} \text{c }=\;\frac{2.97\text{ x }150.1\;}{6300\text{ x }1.0\text{ x }0.1}\;\times \;\text{ΔA }\left(\text{D}-\text{xylose}\right)({\text{gL}}^{-1})\;\\ \text{ c }=\;0.7076\times \;\text{ΔA }\left(\text{D}-\text{xylose}\right)({\text{gL}}^{-1})\; \end{array}$$

$$D-xylose(g/100g)=\frac{\text{C }\left(\text{D}-\text{xylose}\right)\;\left({\text{gL}}^{-1}\text{ sample solution}\right)}{\text{weight }\left(\text{sample}\right)\;\left({\text{gL}}^{-1}\text{ sample solution}\right)}\text{x }100$$

$$Arabinoxylan(g/100g)=\frac{\text{Content of D}-\text{xylose }\left(\text{g}/100\text{ g}\right)\text{x }100}{\left(\text{D}-\text{xylose content of polymer}\right)}$$

### 2.6 Estimation of protein in wheat grains

Total protein content in wheat grains was estimated by Bradford’s protein analysis method (Bradford, 1976). A sample of 100 mg of accurately weighed wheat flour was taken in a 15-ml centrifuge tube and added with 5 ml of Tris–EDTA buffer (pH 7.0). The content was thoroughly mixed and centrifuged at 10,000 rpm for 15 min. In a test tube, 1 ml of supernatant was collected and 3 ml of Bradford’s reagent was added. The absorbance was taken at 595 nm within 1 h, and total protein content was calculated using the BSA standard curve.

### 2.7 Estimation of starch in wheat grains

Total starch content in wheat whole grain flour was estimated by the method described by Chinoy with minor modifications (Chinoy, 1939). A sample of 100 mg of accurately weighed wheat flour was taken in a 15-ml centrifuge tube in which 10 ml of 80% ethanol was added. The content was vigorously mixed and centrifuged at 10,000 rpm for 10 min. The supernatant was discarded, and to the residue 10 ml of 0.7% KOH was added. The mixture was gelatinized for 40 min in a boiling water bath. The content was centrifuged again, and 1 ml aliquot of the supernatant was collected in a test tube in which 1 ml of citrate buffer, 0.5 ml of 20% acetic acid, and 1 ml of I_2_KI_2_ solution were added and solution turned blue-black in colour. The absorbance was measured at 600 nm, and the total starch content was estimated with the standard pure corn starch calibration curve.

### 2.8 Statistical analysis

A statistical analysis of a panel of 478 wheat genotypes for dietary fibre components (β-glucan and arabinoxylan) alongside other grain composition traits including protein, starch, and thousand-grain weight was performed. The analysis was based on pooled observations of the data collected over two consecutive *rabi* seasons (2020–21 and 2021-22) to ensure environmental stability. The descriptive statistics was done using the IBM SPSS statistics version 26. One-way analysis of variance (ANOVA) with Tukey test, Pearson’s correlation, principal component analysis, and heatmap was obtained using OriginPro 2023b. Cluster analysis was performed using the factoextra package of the R Studio v4.1.

## 3 Results and discussion

In this investigation, a panel of 478 wheat genotypes was screened to assess variability in dietary fibre components (β-glucan and arabinoxylan) alongside other grain composition traits such as protein, starch, and thousand-grain weight. The objective was to identify genotypes with superior fibre content that could serve as candidates for food applications and pre-breeding programs.

Details of the genotypes, including their name, β-glucan, arabinoxylan, D-xylose, protein, starch content, and thousand-grain weight, are provided in **Supplementary Table S1**. The overall descriptive statistics for the entire panel—covering wild wheat species, tetraploid, and hexaploid genotypes—are summarized in **Table 1**, which presents the general means, standard deviations, and ranges for each trait.

**Table 1:** Descriptive statistical analysis of a panel of 478 wheat genotypes for dietary fibre components (β-glucan and arabinoxylan) alongside other grain composition traits including protein, starch, and thousand-grain weight.

Measures	β-glucan (% dry wt.)	Arabinoxylan (% dry wt.)	D-xylose (% dry wt.)	Protein (% dry wt.)	Starch (% dry wt.)	Thousand-grain weight (g)
Mean	0.93	5.77	3.58	13.37	68.51	36.74
Standard error	0.02	0.04	0.03	0.12	0.27	0.48
Median	0.84	5.67	3.52	12.85	69.80	38.00
Mode	0.79	5.48	3.40	13.00	71.20	38.00
Standard deviation	0.47	0.87	0.54	2.63	5.93	10.43
Sample variance	0.22	0.76	0.29	6.90	35.16	108.67
Kurtosis	14.79	3.02	2.96	3.12	22.25	0.86
Skewness	3.72	1.18	1.17	1.63	−4.07	−0.88
Range	3.43	6.43	3.98	15.07	60.16	60.55
Sum	444.75	2,757.97	1,710.32	6,390.95	32,746.61	175,63.22
Maximum	3.73	10.34	6.41	24.27	85.33	63.20
Minimum	0.30	3.91	2.43	9.20	25.17	2.65
Confidence level (95.0%)	0.04	0.08	0.05	0.24	0.53	0.94

### 3.1 Distribution of β-glucan in wheat genotypes

The β-glucan content for the entire panel was found in the range of 0.32% (*Ae. diccocoides-*794) to 3.73% (*Ae. peregrina*–629) with a mean value of 0.93%. Among the wild relatives of wheat, the highest and lowest β-glucan contents were found identical with the panel whereas in tetraploid and hexaploid wheat genotypes the highest β-glucan was found in WH-1160 (1.31%) and the lowest in PDW-233 (0.30%) (**Supplementary Table S1**). Pritchard et al. (2011) conducted a similar survey on β-glucan content in 500 wheat accessions, including diploid, tetraploid, and hexaploid genotypes, and reported values ranging from 0.18% to 1.8%. In hexaploid triticale and wheat–barley addition lines, variations in the β-glucan contents were found to be 0.35%–0.96% and 0.90%–1.13%, respectively. The synthetic wheat showed a similar β-glucan content as that of the tetraploid genotypes (Pritchard et al., 2011). It has been also reported that β-glucan in hexaploid wheat was lower than 1% whereas, in tetraploid wheat genotypes, β-glucan content ranged between 0.39% and 0.7%. Among wild relatives of wheat, a high content of β-glucan with a value of up to 7.1% was found in some *Aegilops* species (*Ae. negletta, Ae. umbellulata*, *Ae. biuncialis*, and *Ae. markrafii)* (Marcotuli et al., 2019).

### 3.2 Distribution of arabinoxylan and D-xylose content in wheat genotypes

The arabinoxylan content for the entire panel was found in the range of 3.91% (*T. monococcum*-487) to 10.34% (*Ae. kotschyi*-601) with a mean value of 5.77% whereas the D-xylose content for the whole panel was found in the range of 2.43% (*T. monococcum-487*) to 6.41% (*Ae. kotschyi*-601) with a mean value of 3.58%. Both arabinoxylan and D-xylose traits exhibited a consistent proportional relationship across the genotype groups because D-xylose is the basic unit for arabinoxylan. Therefore, their concentration in wheat genotypes will be equally proportional to each other. Also in the whole panel analysis, among wild wheat genotypes, the highest and lowest arabinoxylan contents and D-xylose content are consistently the same as above. The arabinoxylan content for tetraploid and hexaploid wheat was found to be highest in UP-3043 (7.98%) and lowest in WHD-943 (4.04%). Similarly, D-xylose content was found to be higher in UP-3043 (4.95%) and lowest in WHD-943 (2.50%) (**Supplementary Table S1**). Similar results were found in a survey of wheat accessions including diploid, tetraploid, and hexaploid genotypes, showing a range of arabinoxylan between 2.37% and 10.75%, whereas in wild wheat genotypes, arabinoxylan content was found to be 3.36%–6.92% (Pritchard et al., 2011). The arabinoxylan and xylose content in whole wheat flour of eight different spring wheats ranged 2.93%-4.68% and 23.95%-48.09%, respectively (Saeed et al., 2014). Similarly, in 25 hard spring and 25 hard winter wheat genotypes, total arabinoxylan content was found to range 3.1%–4.0% for winter wheat and 3.9%–4.7% for spring wheat genotypes (Morris et al., 2008).

It also has been reported that the addition of *Aegilops* chromosomes has the potential to increase the β-glucan content and can improve the arabinoxylan content of hexaploid wheat grains. In an investigation, Rakszegi et al. (2017) studied that in all *Aegilops* accessions, the β-glucan content was found higher than the hexaploid wheat genotypes. The β-glucan content varied between 3% and 5% in *Ae. biuncialis* and *Ae. geniculate.* Incorporation of *Ae. biuncialis* chromosomes (3Ub, 2Mb, 3Mb, and 7Mb) had a significant increase in the β-glucan content in the genetic background of Chinese Spring. In contrast, only a small non-significant increase was observed in the total arabinoxylan content of wheat when chromosomes *Ae. geniculate* were added (5Ug and 7Ug) (Rakszegi et al., 2017).

### 3.3 Distribution of protein in wheat genotypes

The protein content for the entire panel was found in the range of 9.20% (PBW-120) to 24.27% (*Ae. peregrina*-13772) with a mean value of 13.37%. Wild relatives of wheat have the highest protein content, whereas within wild relatives, the lowest was found in *Ae. dicoccoides*-794 (15.57%). Among tetraploid and hexaploid wheat genotypes, the highest protein content was found in PDW-291 (19.72%) whereas the lowest was found in PBW-120 (9.20%) (**Supplementary Table S1**). In a similar study, the protein content of 27 different cultivars of wheat was found to range between 13.12% and 19.41% (Csiszár et al., 2010). The protein content in 88 soft red winter wheat varieties was reported to range between 11.7% and 14.8%, with an average of 13% (Bruening, 2010). In one investigation, it was reported that protein content was generally higher in all *Aegilops* accessions compared with hexaploid wheat genotypes. However, considerable variation was observed among the *Aegilops* species themselves. For example, protein content in *Ae. biuncialis* accessions ranged from 22% to 37%, whereas in *Ae. geniculata* genotypes, it was around 26%. The protein content of Chinese Spring wheat was enhanced by the insertion of *Ae. geniculata* chromosomes 2Mg, 5Mg, and 7Mg, as well as 2Ug, 4Ug, 5Ug, and 7Ug (Rakszegi et al., 2017). In a comparative study of 49 emmer (*Triticum turgidum dicoccum*) and 36 einkorn (*Triticum monococcum* L.) wheat genotypes along with durum wheat cultivars (*Triticum turgidum durum*), the protein content varied 13.51%-23.92%, 15.76%-24.75%, and 14.91%-18.15%. Protein levels varied in both emmer and einkorn lines, but no significant difference was observed between emmer and durum wheat (Akar et al., 2019).

Jiang et al. (2008) conducted a comparative study on 17 wild wheat species and three common wheat genotypes. They reported that protein content in the wild species ranged from 13.07% to 19.21%, with a mean of 16.67%, which was 23.21% higher than that of common wheat. Within the wild species, *T. araraticum* had the highest protein content (19.21%), whereas *T. compactum* had the lowest (13.07%). The mean protein levels of diploid (16.91%) and tetraploid (16.84%) genotypes did not differ significantly, whereas hexaploid genotypes showed a lower mean protein content (~16.04%) compared with the other wheat groups. In a similar study by Tran et al. (2020), the average protein content in 26 different wheat varieties was found to be 15.59% in the diploid wheat and 16.04% in tetraploid wheat whereas the lowest protein content was found in common wheat and bread wheat landraces i.e. 12.76%, and 13.15%, respectively.

### 3.4 Distribution of starch in wheat genotypes

The total starch content in the entire panel was found to be varied between a range of 25.16% (*Ae. peregrina*-629) to 85.33% (PDW-274) with a mean value of 68.51%. The highest starch content among wild relatives of wheat was found in *Ae. tauschii*-282 (76.36%) and the lowest was in *Ae. peregrina*-629 (25.16%), whereas in tetraploid and hexaploid wheat, the highest starch content was found in PDW-274 (85.33%) and the lowest was found in Ajanta (61.20%) (**Supplementary Table S1**). In a similar study conducted by Pritchard et al. (2011), the starch content in diploid, tetraploid, and hexaploid genotypes ranged between 31.89% and 83.75%, whereas in ancient wheat genotypes, the starch content ranged from 31.89% to 70.00% (Pritchard et al., 2011). In a similar study conducted on 99 accessions of 20 species of *Aegilops* and 200 accessions of hexaploid wheat, the starch content was found to range 20%-35% and 18%-36%, respectively (Stoddard and Sarker, 2000). Csiszár et al. (2010) studied the starch content of 27 different cultivars of wheat ranging between 60.14% and 66.93% (Csiszár et al., 2010). In another study, the starch content evaluated in 88 soft red winter wheat genotypes was found to range between 66.5% and 69.7%, with an average of 68.2% (Bruening, 2010). Rhazi et al. (2021) reported that the average starch content in 14 French bread cultivars was 61.10%, ranging from 54% to 69.48%, with a low coefficient of variation (CV).

### 3.5 Variation in thousand-grain weight among wheat genotypes

The thousand-grain weight in the entire panel was found to vary between a range of 6.62 g (*Ae.* sp*eltoides-3581*) to 63.20 g (PDW-233) with a mean value of 36.34 g. The highest thousand-grain weight among wild relatives of wheat was found in *Ae. araraticum-4692* (34.56 *g*) and the lowest was in *Ae.* sp*eltoides-3,581* (6.62 *g*), whereas in tetraploid and hexaploid wheat, the highest thousand-grain weight was found in PDW-233 (63.20 g) and the lowest was found in K-7,903 (HALNA) (16.50 g) (**Supplementary Table S1**). Pritchard et al. (2011) surveyed 500 wheat accessions, including diploid, tetraploid, and hexaploid genotypes, to assess grain weight. They reported a wide range, from 3.7 to 78.3 mg across the collection. Within this, 35 ancient wheat lines and 338 hexaploid lines showed grain weights of 3.7–64.6 and 16.2–59.1 mg, respectively. In contrast, tetraploid wheat genotypes displayed grain weights ranging from 20.1 to 74.6 mg. Marcotuli et al. (2019) studied 43 wild and cultivated wheat genotypes (*Triticum* and *Aegilops* species) and reported an average grain weight of 26.5 mg, ranging from 4.1 to 67.4 mg. Within the *Triticum* species, mean grain weight values ranged from 36.6 to 37.8 mg, whereas the *Aegilops* group showed lower mean values, ranging from 11.5 to 13.4 mg.

### 3.6 Analysis of variance and Pearson’s correlation

**Figure 1:**
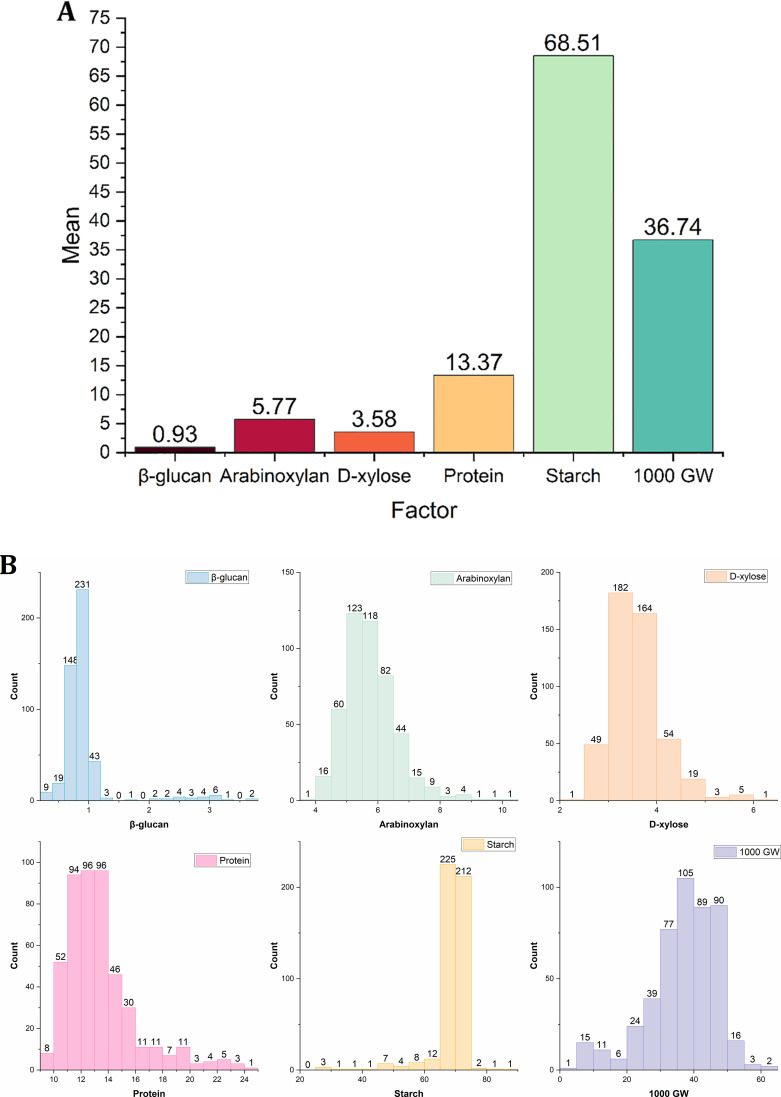
**(A)** Mean concentration and **(B)** frequency distribution of genotypes showing variation in their dietary fibre components (β-glucan and arabinoxylan) alongside other grain composition traits including protein, starch, and thousand-grain weight (1,000 GW) in a whole panel of 478 wheat genotypes.

Among the entire panel, 50 best genotypes were selected for individual parameters as the best candidates to be used in food applications and breeding program (**Supplementary Table S2**). High significant variation (p ≤ 0.001) was observed by analysis of variance (ANOVA) among genotypes for all the factors (**Table 2**). The mean concentration and frequency distribution of genotypes for dietary fibre components (β-glucan and arabinoxylan), along with other grain composition traits such as protein, starch, and thousand-grain weight, were analysed in the full panel of 478 wheat genotypes. The analysis was carried out using OriginPro 2023b, and the results are presented in **Figures 1A, B**, respectively. The mean frequency distribution signifies variation in dietary fibre components (β-glucan and arabinoxylan) alongside other grain composition traits including protein, starch, and thousand grain-weight among wheat genotypes. β-Glucan was skewed toward low values (0.5%-1.5%), whereas arabinoxylan and D-xylose followed near-normal distributions centred around 6%-7% and 3%-4%, respectively. Protein content ranged broadly (10%-24%) with most genotypes between 12% and 15%. Starch content was narrowly distributed (60%-70%), whereas thousand-grain weight exhibited a wider, near-normal distribution (30–45 g).

**Table 2:** Analysis of variance in a panel of 478 wheat genotypes for dietary fibre components (β-glucan and arabinoxylan) alongside other grain composition traits including protein, starch, and thousand grain-weight.

Source	Degree of freedom	Sum of squares	Mean square	F value	Prob>f
Model	5	1,672,950.48	334,590.10	13,207.53	<0.0001
Error	2,862	72,503.87	25.33		
Total	2,867	1,745,454.36			

**Figure 2:**
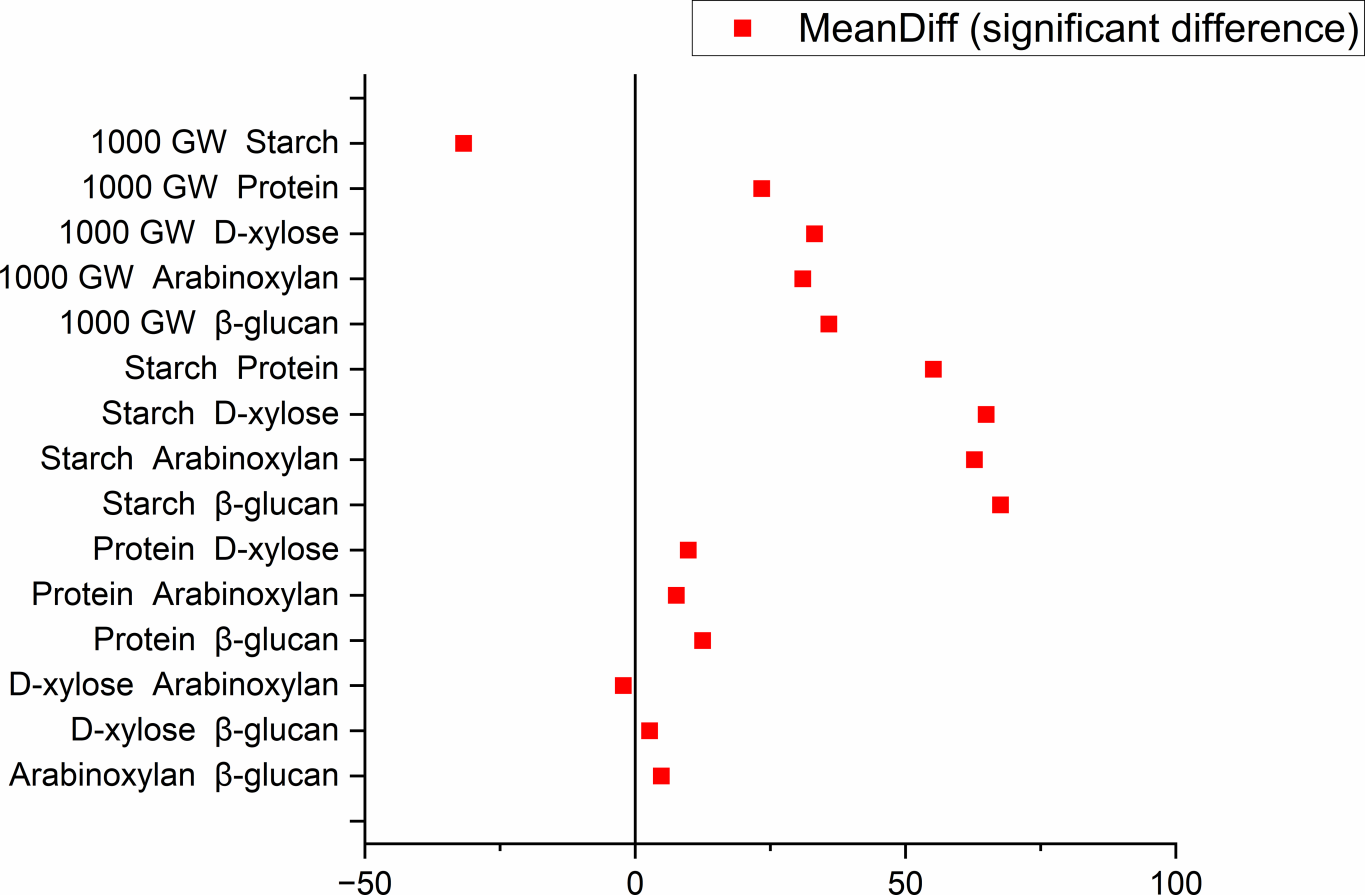
Mean significant differences of dietary fibre components (β-glucan and arabinoxylan) alongside other grain composition traits including protein, starch, and thousand-grain weight (1,000 GW) in a whole panel of 478 wheat genotypes.

A significant negative association (p ≤ 0.001) was observed between thousand-grain weight and starch, whereas no significant associations were observed among β-glucan, arabinoxylan, and D-xylose. A positive significant difference was found among all other quality traits, shown in **Figure 2**. Among all the parameters, thousand grain weight exhibited the highest significant variation and deviation from the median, whereas β-glucan content showed the least variation.

**Figure 3:**
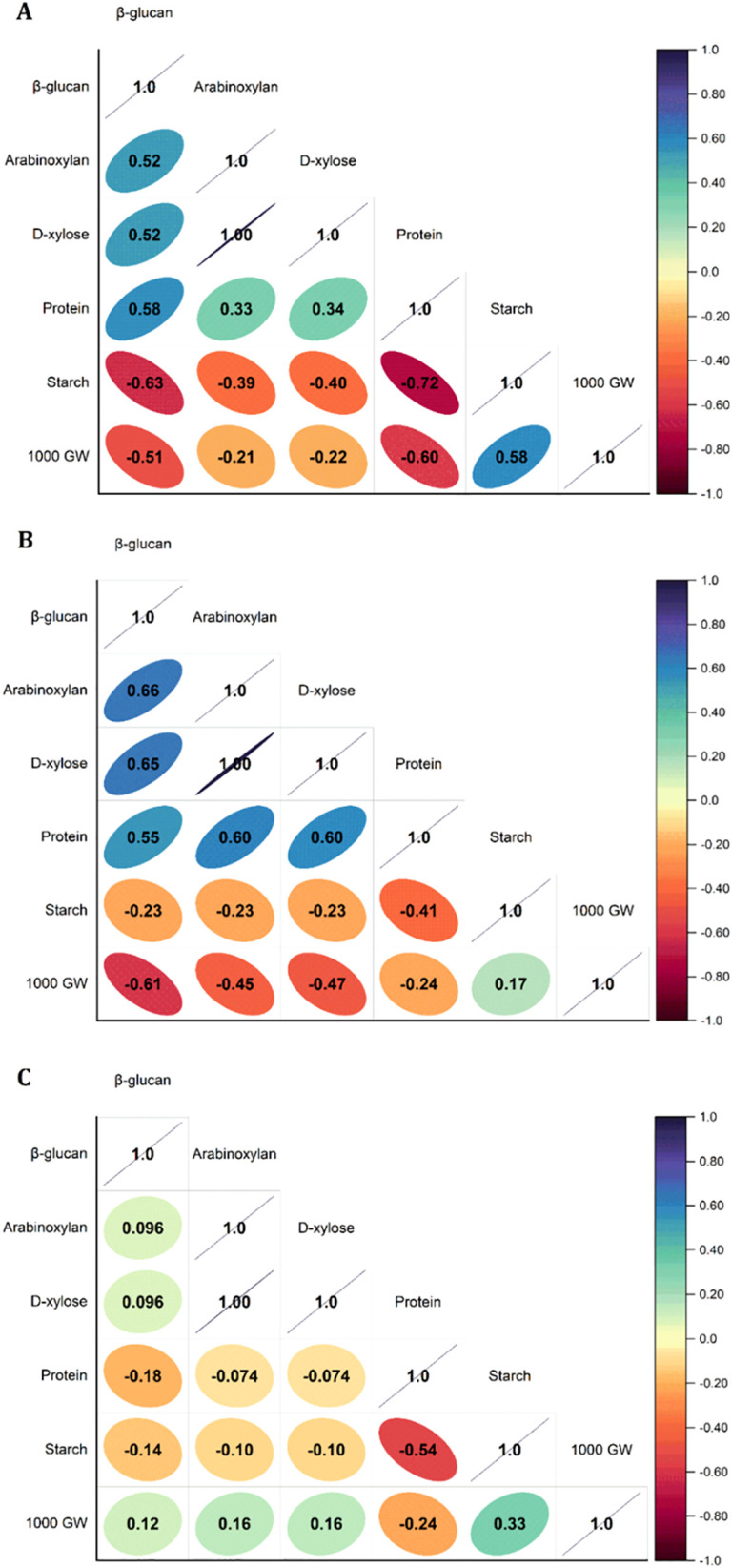
Pearson correlation matrix heat map between dietary fibre components (β-glucan and arabinoxylan) alongside other grain composition traits including protein, starch, and thousand-grain weight (1,000 GW) in **(A)** a whole panel of 478 wheat genotypes, **(B)** wild relatives of wheat genotypes, and **(C)** tetraploid and hexaploid wheat genotypes. A positive and negative correlation is presented by the direction of inclination right tilt and left tilt of eclipse, respectively; correlation coefficients (r) are written in the eclipses and indicated with the size and colour intensity associated with the values.

The interaction between dietary fibre components (β-glucan and arabinoxylan) alongside other grain composition traits including protein, starch, and thousand-grain weight was determined using Pearson’s correlation for indirect selection of the required quality parameters and to find the best genotypes with high β-glucan content and good nutritional values. In the whole panel of wheat genotypes, a significant and positive correlation was observed for β-glucan with arabinoxylan (r = 0.52), D-xylose (r = 0.52), and protein (r = 0.58), whereas with starch and thousand-grain weight, β-glucan shows a highly significant and negative correlation (r = −0.63 and r = −0.51, respectively) (**Figure 3A**). Among wild wheat genotypes, an exactly similar pattern was observed; arabinoxylan, D-xylose, and protein all showed a high positively significant correlation with β-glucan (r = 0.66, r = 0.65, r = 0.55, respectively), whereas starch and thousand-grain weight showed a high negatively significant correlation with β-glucan (r = −0.23, r = −0.61, respectively) (**Figure 3B**). Correlation analysis of tetraploid and hexaploid wheat genotypes revealed a non-significant correlation between dietary fibres. β-Glucan and arabinoxylan both were negatively correlated with starch and protein (r = −0.18 and r = −0.14, respectively), whereas a least positive correlation was found between dietary fibres and thousand-grain weight (r = 0.12, on average) (**Figure 3C**). Protein and starch were found to be highly significant and negatively correlated, whereas starch and thousand-grain weight were found highly significant positively correlated in all datasets. Arabinoxylan and D-xylose showed a positive and highly significant correlation in all datasets. Pritchard et al. (2011) investigated 500 wheat accessions, including diploid, tetraploid, and hexaploid genotypes, and reported no relationships between β-glucan, arabinoxylan, and total starch content. However, they observed a strong correlation between grain weight and total starch. These findings are consistent with previous studies showing a negative relationship between starch and dietary fibre content in wheat, suggesting a potential trade-off between carbohydrate storage and health-benefiting polysaccharides.

In a study by Csiszár et al. (2010), it has been reported that in wheat grains, starch molecules are contained in the endosperm within a protein matrix and a negative correlation was found between the protein and starch contents of 27 different cultivars of wheat. Also, it has been observed that genotypes with high starch content relatively possess low grain protein content because starch and protein contents are negatively correlated (Csiszár et al., 2010). In another study by Rakszegi et al. (2017), it has been reported that the amount of protein and β-glucan in all *Aegilops* accessions was significantly higher than the hexaploid wheat genotypes. Therefore, the insertion of the *Aegilops* chromosome enhanced the amount of protein and β-glucan in common wheat, which can contribute to the “yield dilution” effect. Additionally, starch content of wheat increased whereas its grain storage proteins and β-glucan concentration decreased (Rakszegi et al., 2017). Marcotuli et al. (2019) reported a negative and significant correlation (r = –0.34) between grain weight and β-glucan content in a panel of wild, tetraploid, and hexaploid wheat genotypes. Their investigation also showed that cultivars with high starch levels tended to have comparatively low β-glucan and protein contents.

### 3.7 Principal component analysis

**Figure 4:**
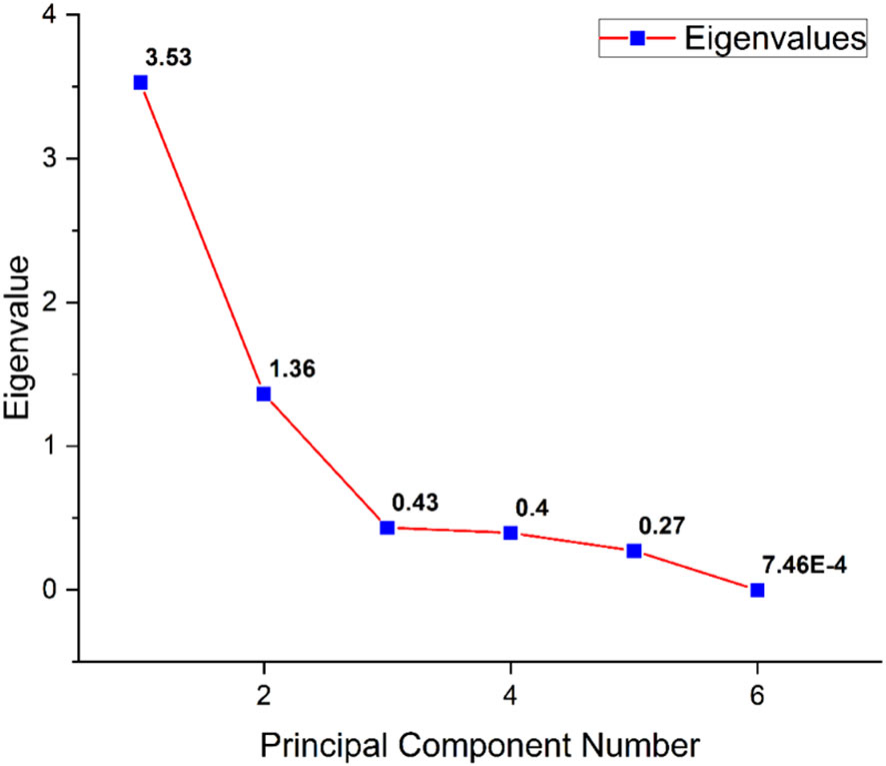
Scree plot of principal component analysis (PCA) showing the contribution of each principal component (PC) for a panel of 478 wheat genotypes representing six principle components and their eigenvalues depicting that only PC1 and PC2 are statistically significant.

Principal component analysis was performed for six parameters of wheat, and among the six principal components, PC1 and PC2 accounted for the majority of total variance observed (**Figure 4**). Most of the variability, 81.59%, was described by the first two principal components (PCs) (58.84% and 22.74%, respectively) (**Table 3**). Therefore, the two PCs were used to construct the PC biplot. PC1 showed a strong positive association with β-glucan and protein, reflecting their significant positive correlation. In contrast, PC2 was negatively associated with starch and thousand-grain weight, although these two traits were strongly and positively correlated with each other, whereas a negative association was found with starch and thousand-grain weight between each of the following: β-glucan, arabinoxylan, D-xylose, and protein. The whole panel was assembled into two groups, one comprising all the wild relatives of wheat and the other one having all the tetraploid and hexaploid wheat genotypes.

**Table 3:** Eigenvalue, percentage of total variation, and cumulative percentage of six principal components for parameters, *viz.*, β-glucan, arabinoxylan, D-xylose, protein, starch, and thousand-grain weight of 478 wheat genotypes.

Principal Components	Eigenvalue	Variability (%)	Cumulative %
PC1	3.53	58.84	58.84
PC2	1.36	22.74	81.59
PC3	0.43	7.24	88.83
PC4	0.40	6.62	95.45
PC5	0.27	4.54	99.99
PC6	0.00	0.01	100.00

**Figure 5:**
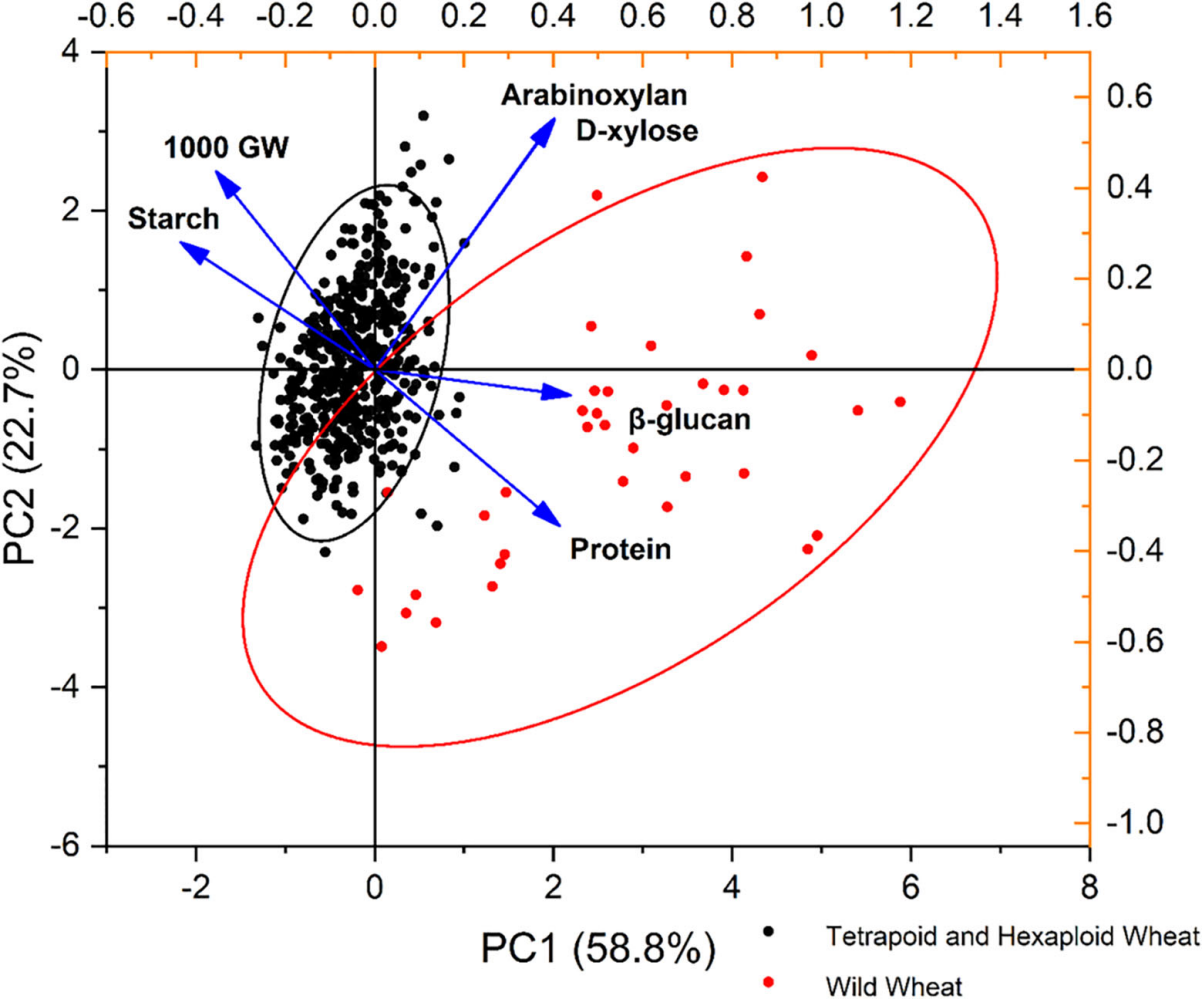
Principal component analysis (PCA) bi-plot of a panel of 478 wheat genotypes with dietary fibre components (β-glucan and arabinoxylan) alongside other grain composition traits including protein, starch, and thousand-grain weight (1,000 GW) (explanatory variables, n = 6). The bi-plot shows the PCA scores of the explanatory variables as vectors (in black) and wheat genotypes (i.e., acoustic marks) of two groups wild relatives of wheat (red dots) and tetraploid and hexaploid wheat genotypes (black dots) of the first (x-axis) and second (y-axis) principal components (PCs). Genotypes on the same side of the variable are having a high contribution on it. The magnitude of the vectors (lines) shows the strength of their contribution to each PC. Vectors pointing in similar directions indicate positively correlated variables, vectors pointing in opposite directions indicate negatively correlated variables, and vectors at proximately right angles indicate low or no correlation. Coloured ellipses show the marked observations grouped as wild relatives of wheat (red eclipse) and tetraploid and hexaploid wheat genotypes (black eclipse).

All wild wheat genotypes clustered along PC1, characterized by high grain β-glucan and protein content. In contrast, the tetraploid and hexaploid wheat genotypes were clumped near the centre of the biplot, indicating less variation among these groups for the studied parameters (**Figure 5**). Shewry et al. (2013) conducted a study on 150 bread wheat genotypes, classifying them through principal component analysis based on differences in dietary fibre, starch, and protein contents. They reported a negative correlation between starch and protein, indicating that genotypes with lower starch levels tend to have higher grain protein content. Such multivariate analyses are valuable for classifying and selecting superior genotypes that can be used in breeding programs targeting these traits.

### 3.8 Cluster analysis and heat map

**Figure 6:**
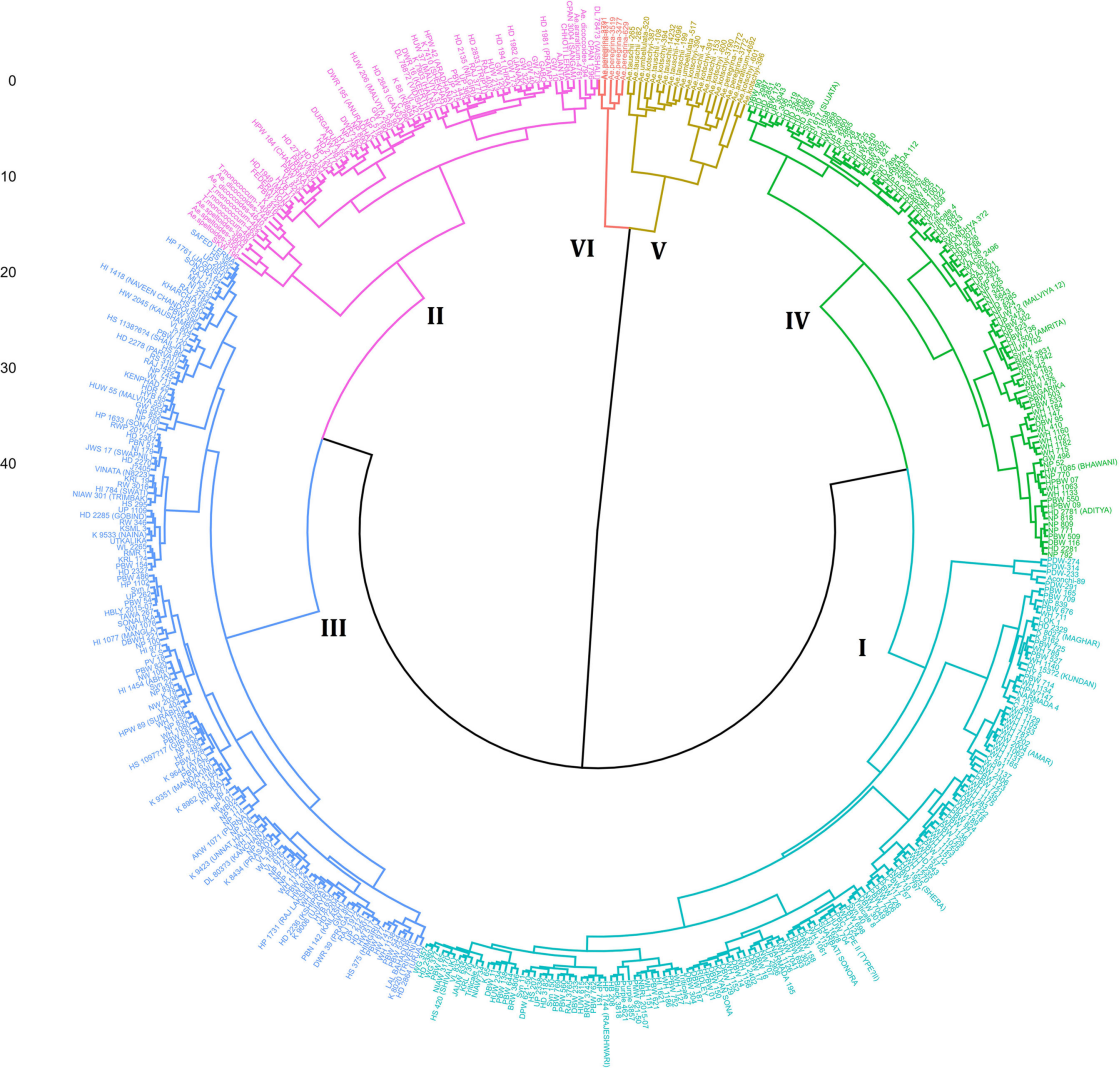
Dendrogram of 478 wheat genotypes for the dietary fibre components (β-glucan and arabinoxylan) alongside other grain composition traits including protein, starch, and thousand-grain weight (1,000 GW). The genotypes were divided into six main clusters in the dendrogram on the basis of different concentrations in their biochemical parameters representing the genetic variability among the genotypes, i.e., cluster I (pink), cluster II (blue), cluster III (green), cluster IV (sky blue), cluster V (light brown), and cluster VI (red).

Multivariate analysis was conducted on the entire panel of 478 genotypes for dietary fibre components (β-glucan and arabinoxylan), along with other grain composition traits such as protein, starch, and thousand-grain weight. This analysis evaluated variation in trait concentrations among wheat genotypes and established the detectable ranges in this collection, as shown in **Supplementary Figure S1**. Cluster analysis grouped the genotypes into six major clusters, primarily based on fibre content and grain composition traits. This categorization provides valuable insights into the genetic diversity and potential of specific accessions for trait-based selection in breeding programs. The dendrogram separated the genotypes into six major clusters, i.e., cluster I (pink), cluster II (blue), cluster III (green), cluster IV (sky blue), cluster V (light brown), and cluster VI (red) (**Figure 6**), and their cluster mean is presented in **Table 4**.

**Table 4:** Cluster mean for dietary fibre components (β-glucan and arabinoxylan) alongside other grain composition traits including protein, starch, and thousand-grain weight in a panel of 478 wheat genotypes.

Cluster	Number of genotypes	β-glucan	Arabinoxylan	D-xylose	Protein	Starch	Thousand-grain weight
C1	170	0.81	5.07	3.14	12.93	70.18	36.53
C2	61	0.73	5.66	3.51	15.85	64.69	27.31
C3	68	0.88	6.75	4.18	12.47	69.48	44.25
C4	152	0.86	5.81	3.60	11.97	70.44	41.94
C5	22	2.50	7.63	4.75	20.31	58.68	11.23
C6	5	3.36	8.31	5.15	22.44	29.38	11.06

**Figure 7:**
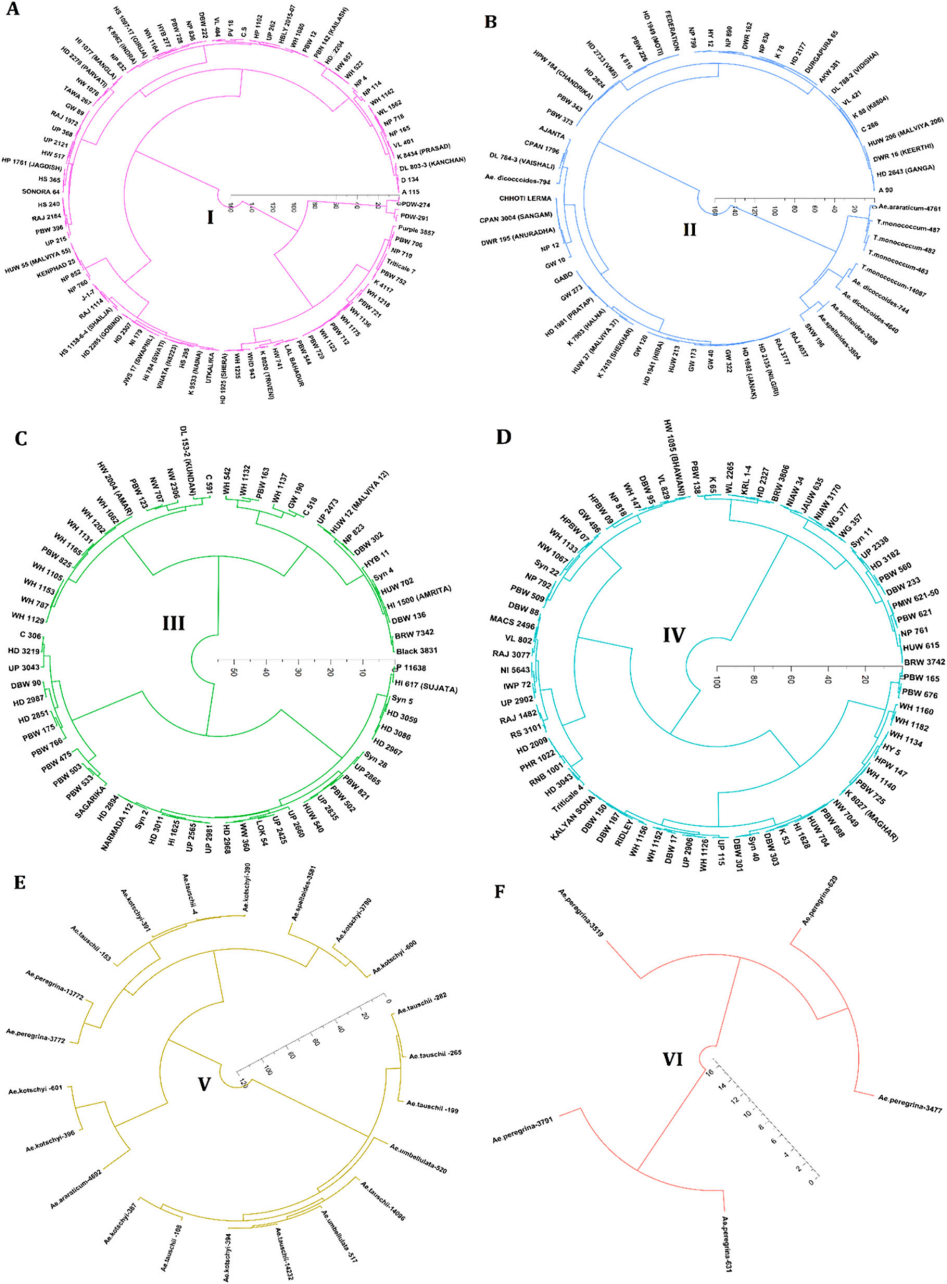
Individual clusters of dendrogram of 478 wheat genotypes based on their dietary fibre components (β-glucan and arabinoxylan) alongside other grain composition traits including protein, starch, and thousand-grain weight (1,000 GW). **(A)** Cluster I (pink), **(B)** cluster II (blue), **(C)** cluster III (green), **(D)** cluster IV (sky blue), **(E)** cluster V (light brown), and **(F)** cluster VI (red) consisted of 170, 61, 68, 152, 22, and 5 wheat genotypes, respectively.

Cluster I is the largest cluster with 170 genotypes and had the lowest cluster mean for β-glucan and arabinoxylan content and the highest cluster mean for starch content (**Figure 7A**). Cluster II consisted of 61 genotypes, exceptionally having a high cluster mean for starch content compared with other clusters and lower cluster mean for β-glucan content (**Figure 7B**). Cluster III containing 68 genotypes consisting of hexaploid wheat varieties exceptionally no wild wheat genotype was found in this cluster with a high cluster mean for thousand-grain weight (**Figure 7C**). Cluster IV consisted of 152 wheat genotypes with the lowest mean for protein content and highest for starch and thousand-grain weight (**Figure 7D**). Cluster V consisted of 22 genotypes with most of the wild relatives of wheat specially *Aegilops* species with high β-glucan content and lower starch content (**Figure 7E**). Cluster VI, the smallest with five wheat genotypes with only *Ae. peregrina* genotypes, has the highest cluster mean for β-glucan, arabinoxylan, and protein content and the lowest cluster mean for starch content and thousand-grain weight (**Figure 7F**). Primitive grain species with small seeds showed the highest variation in β-glucan content, indicating that an increase in this important grain constituent, β-glucan, can significantly enhance the nutritional profile of wheat (Pritchard et al., 2011). Genotypes such as WL 1562, PDW-314, HI 1625, PBW475, HI 1628, BRW3806, *Ae. tauschii-4*, *Ae. peregrina-3477*, and *Ae. peregrina-631* were selected based on their combined high levels of β-glucan, arabinoxylan, and protein, as well as agronomically favourable traits such as higher thousand grain weight. These genotypes demonstrate superior nutritional profiles and potential for pre-breeding for improving dietary fibre content in wheat. The genotypes in clusters V and VI may be utilized for improving the β-glucan, arabinoxylan, and protein content, whereas clusters I and IV may be utilized for improving the starch content in wheat genotypes. These selected genotypes provide a valuable starting point for trait introgression and the development of nutrient-dense wheat varieties.

## 4 Conclusion

The potential of wild genetic resources to increase the amount of β-glucan in wheat grain has only been briefly described in a few studies. The finding of the present investigation on a panel of 478 different wheat genotypes revealed the variability in their dietary fibre, starch, and protein content. A highly positive and significant correlation was found between dietary fibres and protein whereas a negative correlation was found with their starch and thousand-grain weight. This study suggested that *Aegilops* species could be a potential genetic resource for improving dietary fibre and protein content and designated as the best candidates with enhanced β-glucan content for well-being and prevention of major health issues. The differences in concentration of dietary fibre content among these 478 wheat genotypes revealed their genetic variability and therefore could be assigned in different clusters with their best representative genotypes WL 1562, PDW-314, HI 1625, PBW475, HI 1628, BRW3806, *Ae. tauschii-4*, *Ae. peregrina-3477*, and *Ae. peregrina-631*. These selected genotypes can be further enhanced through backcrossing or by incorporating them into cultivated wheat via intergeneric and interspecific hybridization. Such approaches enable the development of chromosome introgression, addition, or substitution lines, which can subsequently be exploited in marker-assisted selection (MAS) programmes to identify key genes and QTLs associated with dietary fibres, particularly β-glucan. The genotype selection and prominent nutritional attributes based upon the present study will provide a solid platform for future crop improvement strategies to produce superior wheat cultivars with high β-glucan content and yield for food and nutritional security.

## Data Availability

The original contributions presented in the study are included in the article/**Supplementary Material**, further inquiries can be directed to the corresponding author.

## References

[B1] Akar T. Cengiz M. F. Tekin M. (2019). A comparative study of protein and free amino acid contents in some important ancient wheat lines. Qual. Assur. Saf. Crop Foods. 11, 191–200. doi: 10.3920/QAS2018.1382

[B2] Bingham S. A. Day N. E. Luben R. Ferrari P. Slimani N. Norat T. . (2003). Dietary fibre in food and protection against colorectal cancer in the European Prospective Investigation into Cancer and Nutrition (EPIC): An observational study. Lancet 361, 1496–1501. doi: 10.1016/S0140-6736(03)13174-1, PMID: 12737858

[B3] Bradford M. M. (1976). A rapid and sensitive method for the quantitation of microgram quantities of protein utilizing the principle of protein-dye binding. Anal. Biochem. 72, 248–254. doi: 10.1016/0003-2697(76)90527-3, PMID: 942051

[B4] Bruening B. (2010). Varietial differences in wheat grain starch, protein and fiber content (Lexington, KY, USA: Plant & Soil Sciences Department, University of Kentucky), 1–3.

[B5] Chinoy J. J. (1939). A new colorimetric method for the determination of starch applied to soluble starch, natural starches, and flour. Mikrochim. Acta 26, 132–142. doi: 10.1007/BF01403036

[B6] Collins H. M. Burton R. A. Topping D. L. Liao M. L. Bacic A. Fincher G. B. (2010). Variability in fine structures of noncellulosic cell wall polysaccharides from cereal grains: Potential importance in human health and nutrition. Cereal Chem. 87, 272–282. doi: 10.1094/CCHEM-87-4-0272

[B7] Crowe F. L. Appleby P. N. Allen N. E. Key T. J. (2011). Diet and risk of diverticular disease in Oxford cohort of European Prospective Investigation into Cancer and Nutrition (EPIC): Prospective study of British vegetarians and non-vegetarians. BMJ 343, 1–15. doi: 10.1136/bmj.d4131, PMID: 21771850 PMC3139912

[B8] Csiszár J. Guóth A. Kolbert Z. Gallé Á. Tari I. Ciulca S. . (2010). Starch to protein ratio and α-amylase activities in grains of different wheat cultivars. Acta Biol. Szeged. 54, 19–23.

[B9] Dona A. C. Pages G. Gilbert R. G. Kuchel P. W. (2010). Digestion of starch: *In vivo* and *in vitro* kinetic models used to characterise oligosaccharide or glucose release. Carbohydr. Polym. 80, 599–617. doi: 10.1016/j.carbpol.2010.01.002

[B10] EFSA Panel on Dietetic Products Nutrition and Allergies (2010). Scientific Opinion on the substantiation of a health claim related to oat beta glucan and lowering blood cholesterol and reduced risk of (coronary) heart disease pursuant to Article 14 of Regulation (EC) No 1924/2006. EFSA. J. 8, 1–15. doi: 10.2903/j.efsa.2010.1885 PMC13085209 42004118

[B11] FAO (2022). FAOSTAT Statistical Database (Rome, Italy: Food and Agriculture Organization of the United Nations, 1997). Available online at: https://www.fao.org/faostat/en/data.

[B12] Gebruers K. Dornez E. Bedõ Z. Rakszegi M. Frás A. Boros D. . (2010). Environment and genotype effects on the content of dietary fiber and its components in wheat in the HEALTHGRAIN diversity screen. J. Agric. Food Chem. 58, 9353–9361. doi: 10.1021/jf100447g, PMID: 20462191

[B13] Giancaspro A. Giove S. L. Zito D. Blanco A. Gadaleta A. (2016). Mapping QTLs for Fusarium head blight resistance in an interspecific wheat population. Front. Plant Sci. 7. doi: 10.3389/fpls.2016.01381, PMID: 27746787 PMC5040704

[B14] Havrlentová M. Kraic J. (2006). Content of β-D-glucan in cereal grains. J. Food Nutr. Res. 45, 97–103.

[B15] Jiang X.l. Tian J.c. Hao Z. Zhang W.d. (2008). Protein content and amino acid composition in grains of wheat-related species. Agric. Sci. China 7, 272–279. doi: 10.1016/S1671-2927(08)60066-8

[B16] Kumar U. Mathpal P. Malik S. Kumar N. (2016). Evaluation of iron and zinc in grain and grain fractions of hexaploid wheat and its related species for possible utilization in wheat biofortification. Plant Genet. Resour. Charact. Util. 14, 101–111. doi: 10.1017/S147926211500012X

[B17] Lafiandra D. Riccardi G. Shewry P. R. (2014). Improving cereal grain carbohydrates for diet and health. J. Cereal Sci. 59, 312–326. doi: 10.1016/j.jcs.2014.01.001, PMID: 24966450 PMC4064937

[B18] Lattimer J. M. Haub M. D. (2010). Effects of dietary fiber and its components on metabolic health. Nutrients 2, 1266–1289. doi: 10.3390/nu2121266, PMID: 22254008 PMC3257631

[B19] Limberger-Bayer V. M. De Francisco A. Chan A. Oro T. Ogliari P. J. Barreto P. L. M. (2014). Barley β-glucans extraction and partial characterization. Food Chem. 154, 84–89. doi: 10.1016/j.foodchem.2013.12.104, PMID: 24518319

[B20] Lunn J. Buttriss J. L. (2007). Carbohydrates and dietary fibre. Nutr. Bull. 32, 21–64. doi: 10.1111/j.1467-3010.2007.00616.x

[B21] Malunga L. N. Izydorczyk M. Beta T. (2017). Antiglycemic effect of water extractable arabinoxylan from wheat aleurone and bran. J. Nutr. Metab. 2017, 1–6. doi: 10.1155/2017/5784759, PMID: 28626590 PMC5463155

[B22] Marcotuli I. Colasuonno P. Cutillo S. Simeone R. Blanco A. Gadaleta A. (2019). β-glucan content in a panel of Triticum and Aegilops genotypes. Genet. Resour. Crop Evol. 66, 897–907. doi: 10.1007/s10722-019-00753-1

[B23] Marcotuli I. Colasuonno P. Hsieh Y. S. Y. Fincher B. (2020). Non-starch polysaccharides in durum wheat: A review. Int. J. Mol. Sci. 21, 1–18. doi: 10.3390/ijms21082933, PMID: 32331292 PMC7215680

[B24] Mares D. J. Stone B. A. (1973). Studies on wheat endosperm II. Properties of the wall components and studies on their organization in the wall. Aust. J. Biol. Sci. 26, 813–830. doi: 10.1071/BI9730813

[B25] Morris C. F. Li S. Bettge A. D. King G. E. Garland-Campbell K. Gill K. S . (2008). “ Arabinoxylan content of hard winter and spring wheats of the US Pacific Northwest,” in The 11th International Wheat Genetics Symposium proceedings Edited by Rudi Appels, Russell Eastwood, Evans Lagudah, Peter Langridge, Michael Mackay, Lynne McIntyre, and Peter Sharp ( Sydney University Press, Sydney), 1–3. Available online at: http://hdl.handle.net/2123/3349 (Accessed October 09, 2025).

[B26] Nirmala Prasadi V. P. Joye I. J. (2020). Dietary fibre from whole grains and their benefits on metabolic health. Nutrients 12, 1–20. doi: 10.3390/nu12103045, PMID: 33027944 PMC7599874

[B27] Pritchard J. R. Lawrence G. J. Larroque O. Li Z. Laidlaw H. K. C. Morell M. K. . (2011). A survey of β-glucan and arabinoxylan content in wheat. J. Sci. Food Agric. 91, 1298–1303. doi: 10.1002/jsfa.4316, PMID: 21469147

[B28] Rakszegi M. Molnár I. Lovegrove A. Darkó É. Farkas A. Láng L. . (2017). Addition of Aegilops U and M chromosomes affects protein and dietary fiber content of wholemeal wheat flour. Front. Plant Sci. 8. doi: 10.3389/fpls.2017.01529, PMID: 28932231 PMC5592229

[B29] Rhazi L. Benoît M. Daaloul O. Branlard G. Aussenac T. (2021). Genetic and environmental variation in starch content, starch granule distribution and starch polymer molecular characteristics of French bread wheat. Foods 10, 205. doi: 10.3390/foods10020205, PMID: 33498368 PMC7909431

[B30] Saeed F. Pasha I. Anjum F. M. Sultan J. I. Arshad M. (2014). Arabinoxylan and arabinogalactan content in different spring wheats. Int. J. Food Prop. 17, 713–721. doi: 10.1080/10942912.2012.654568

[B31] Saulnier L. Sado P.-E. Branlard G. Charmet G. Guillon F. (2007). Wheat arabinoxylans: Exploiting variation in amount and composition to develop enhanced varieties. J. Cereal Sci. 46, 261–281. doi: 10.1016/j.jcs.2007.06.014

[B32] Schneider A. Molnár I. Molnár-Láng M. (2008). Utilisation of Aegilops (goat grass) species to widen the genetic diversity of cultivated wheat. Euphytica 163, 1–19. doi: 10.1007/s10681-007-9624-y

[B33] Shewry P. R. Hawkesford M. J. Piironen V. Lampi A. M. Gebruers K. Boros D. . (2013). Natural variation in grain composition of wheat and related cereals. J. Agric. Food Chem. 61, 8295–8303. doi: 10.1021/jf3054092, PMID: 23414336

[B34] Shewry P. R. Hey S. J. (2015). The contribution of wheat to human diet and health. Food Energy Secur. 4, 178–202. doi: 10.1002/FES3.64, PMID: 27610232 PMC4998136

[B35] Stephen A. M. Champ M. M. J. Cloran S. J. Fleith M. Van Lieshout L. Mejborn H. . (2017). Dietary fibre in Europe: Current state of knowledge on definitions, sources, recommendations, intakes and relationships to health. Nutr. Res. Rev. 30, 149–190. doi: 10.1017/S095442241700004X, PMID: 28676135

[B36] Stoddard F. L. Sarker R. (2000). Characterization of starch in Aegilops species. Cereal Chem. 77, 445–447. doi: 10.1094/CCHEM.2000.77.4.445

[B37] Tran K. D. Konvalina P. Capouchova I. Janovska D. Lacko-Bartosova M. Kopecky M. . (2020). Comparative study on protein quality and rheological behavior of different wheat species. Agronomy 10, 1763. doi: 10.3390/agronomy10111763

[B38] Wood P. J. (2007). Cereal β-glucans in diet and health. J. Cereal Sci. 46, 230–238. doi: 10.1016/j.jcs.2007.06.012

