# Supplementary material for: Biofortification of dietary fibre: exploring enhanced β-glucan and arabinoxylan content in a panel of Triticum and wild relatives
Source: Front Plant Sci. 2026 Jan 22;16:1660594. doi: 10.3389/fpls.2025.1660594 (PMC12872903; doi:10.3389/fpls.2025.1660594)
Supplement: Supplementary file 1 [file DataSheet1.docx]

**Biofortification of dietary fibre: Exploring enhanced β-Glucan and arabinoxylan content in a panel of *Triticum* and wild relatives**

**Prexha Kapoor^1^, Sourav Panigrahi^1^, Rita Goswami^1^, Sonu Langaya^2^, Yogita Singh^3^, Sundip Kumar^4^, Krishna Pal Singh^5&6^, Farkhandah Jan^7^, Ivica Djalovic^8^, Reyazul Rouf Mir^7^, Upendra Kumar^1,9^***

^1^Department of Molecular Biology and Biotechnology, College of Biotechnology, Chaudhary Charan Singh Haryana Agricultural University, Hisar, Haryana, 125004, India

^2^Department of Genetics and Plant Breeding, College of Agriculture, Chaudhary Charan Singh Haryana Agricultural University, Hisar, Haryana, 125004, India

^3^Stockbridge School of Agriculture, University of Massachusetts Amherst, MA, 01003, USA

^4^Molecular Cytogenetics Laboratory, Department of Molecular Biology & Genetic Engineering, College of Basic Sciences & Humanities, GB Pant University of Agriculture & Technology, Pantnagar, 263145, India

^5^Biophysics Unit, College of Basic Sciences & Humanities, GB Pant University of Agriculture & Technology, Pantnagar, 263145, India

^6^Vice-Chancellor’s Secretariat, Mahatma Jyotiba Phule Rohilkhand University, Bareilly -243001, India

^7^Division of Genetics and Plant Breeding, Sher-e-Kashmir University of Agricultural Sciences and Technology of Kashmir (SKUAST-Kashmir), Srinagar (J& K), India

^8^Institute of Field and Vegetable Crops, National Institute of the Republic of Serbia, Maxim Gorki, Novi Sad, Serbia

^9^Department of Plant Science, Mahatma Jyotiba Phule Rohilkhand University, Bareilly 243001, India

*Corresponding Authors:

Dr. Upendra Kumar, Email: [baliyan.upendra@gmail.com](mailto:baliyan.upendra@gmail.com)

**Table S1.** List of a panel of 478 wheat genotypes including their accession number, source, country and genotype group with different concentrations in their **dietary fibre components (β-glucan and arabinoxylan) alongside other grain composition traits including protein, starch, and thousand-grain weight**.

| **Sr.** | **Genotype** | **Accession No.** | **Source** | **Country** | **Genotype group** | **β-glucan (% dry wt.)** | **Arabinoxylan (% dry wt.)** | **D-xylose (% dry wt.)** | **Protein (% dry wt.)** | **Starch (% dry wt.)** | **Thousand- Grain Weight (g)** |
| --- | --- | --- | --- | --- | --- | --- | --- | --- | --- | --- | --- |
| 1 | A 115 | NA | CCS HAU | India | Hexaploid | 0.67 | 5.36 | 3.33 | 15.30 | 69.33 | 43.40 |
| 2 | A 90 | NA | CCS HAU | India | Hexaploid | 0.78 | 6.11 | 3.79 | 15.30 | 66.80 | 34.70 |
| 3 | AJANTA | NA | CCS HAU | India | Hexaploid | 0.81 | 5.81 | 3.60 | 18.87 | 61.17 | 26.50 |
| 4 | AKW 1071 (PURNA) | NA | CCS HAU | India | Hexaploid | 0.35 | 5.11 | 3.17 | 14.90 | 67.20 | 35.20 |
| 5 | AKW 381 | NA | CCS HAU | India | Hexaploid | 0.80 | 5.80 | 3.59 | 14.73 | 68.80 | 36.00 |
| 6 | Black 3818 | NA | CCS HAU | India | Hexaploid | 0.77 | 5.23 | 3.24 | 11.83 | 67.33 | 41.40 |
| 7 | Black 3831 | NA | CCS HAU | India | Hexaploid | 0.77 | 6.67 | 4.13 | 11.77 | 66.50 | 42.70 |
| 8 | BRW 3742 | NA | CCS HAU | India | Hexaploid | 0.73 | 5.38 | 3.33 | 11.57 | 71.80 | 47.30 |
| 9 | BRW 3806 | NA | CCS HAU | India | Hexaploid | 0.73 | 4.92 | 3.05 | 9.80 | 73.00 | 44.40 |
| 10 | BRW 7342 | NA | CCS HAU | India | Hexaploid | 0.64 | 6.79 | 4.21 | 11.90 | 68.50 | 38.64 |
| 11 | C 285 | NA | CCS HAU | India | Hexaploid | 0.76 | 5.47 | 3.39 | 15.97 | 67.67 | 40.10 |
| 12 | C 286 | NA | CCS HAU | India | Hexaploid | 0.74 | 5.67 | 3.52 | 16.10 | 67.80 | 36.10 |
| 13 | C 306 | NA | CCS HAU | India | Hexaploid | 0.88 | 7.61 | 4.72 | 10.30 | 71.20 | 46.60 |
| 14 | C 518 | NA | CCS HAU | India | Hexaploid | 0.76 | 6.55 | 4.06 | 16.43 | 66.60 | 38.00 |
| 15 | C 591 | NA | CCS HAU | India | Hexaploid | 0.97 | 6.15 | 3.81 | 16.97 | 66.83 | 48.70 |
| 16 | C.S | NA | CCS HAU | India | Hexaploid | 0.77 | 5.58 | 3.46 | 12.30 | 67.30 | 36.30 |
| 17 | CHHOTI LERMA | NA | CCS HAU | India | Hexaploid | 0.81 | 6.39 | 3.96 | 17.30 | 64.40 | 25.00 |
| 18 | CPAN 1796 | NA | CCS HAU | India | Hexaploid | 0.73 | 5.39 | 3.34 | 17.20 | 65.90 | 27.40 |
| 19 | CPAN 3004 (SANGAM) | NA | CCS HAU | India | Hexaploid | 0.78 | 6.05 | 3.75 | 17.50 | 63.30 | 30.80 |
| 20 | D 134 | NA | CCS HAU | India | Hexaploid | 0.82 | 5.42 | 3.36 | 15.20 | 69.50 | 35.00 |
| 21 | DBW 11 | NA | IIWBR | India | Hexaploid | 0.96 | 5.06 | 3.13 | 10.90 | 73.80 | 47.20 |
| 22 | DBW 116 | NA | IIWBR | India | Hexaploid | 0.77 | 6.27 | 3.88 | 13.00 | 70.00 | 34.54 |
| 23 | DBW 129 | NA | IIWBR | India | Hexaploid | 0.94 | 6.07 | 3.76 | 11.10 | 72.10 | 46.04 |
| 24 | DBW 136 | NA | IIWBR | India | Hexaploid | 0.87 | 6.87 | 4.26 | 13.00 | 68.70 | 37.60 |
| 25 | DBW 14 | NA | IIWBR | India | Hexaploid | 1.02 | 5.81 | 3.60 | 12.13 | 70.70 | 48.20 |
| 26 | DBW 150 | NA | IIWBR | India | Hexaploid | 0.95 | 6.17 | 3.83 | 12.13 | 70.83 | 46.00 |
| 27 | DBW 16 | NA | IIWBR | India | Hexaploid | 0.82 | 5.93 | 3.68 | 12.10 | 71.60 | 49.20 |
| 28 | DBW 17 | NA | IIWBR | India | Hexaploid | 0.87 | 6.06 | 3.76 | 12.53 | 70.60 | 51.70 |
| 29 | DBW 187 | NA | IIWBR | India | Hexaploid | 0.88 | 5.95 | 3.69 | 11.70 | 70.47 | 46.30 |
| 30 | DBW 222 | NA | IIWBR | India | Hexaploid | 1.10 | 5.24 | 3.25 | 13.30 | 69.83 | 41.00 |
| 31 | DBW 233 | NA | IIWBR | India | Hexaploid | 0.95 | 5.75 | 3.57 | 11.03 | 71.20 | 48.70 |
| 32 | DBW 301 | NA | IIWBR | India | Hexaploid | 0.70 | 6.03 | 3.74 | 12.47 | 70.90 | 43.90 |
| 33 | DBW 302 | NA | IIWBR | India | Hexaploid | 0.90 | 6.57 | 4.08 | 12.17 | 69.57 | 40.50 |
| 34 | DBW 303 | NA | IIWBR | India | Hexaploid | 1.03 | 5.60 | 3.47 | 11.90 | 70.10 | 42.14 |
| 35 | DBW 304 | NA | IIWBR | India | Hexaploid | 0.97 | 5.93 | 3.68 | 11.87 | 70.23 | 46.70 |
| 36 | DBW 88 | NA | IIWBR | India | Hexaploid | 0.85 | 6.06 | 3.76 | 10.90 | 71.00 | 40.10 |
| 37 | DBW 90 | NA | IIWBR | India | Hexaploid | 0.85 | 7.32 | 4.54 | 12.63 | 69.33 | 45.60 |
| 38 | DBW 95 | NA | IIWBR | India | Hexaploid | 1.13 | 6.13 | 3.80 | 14.20 | 67.40 | 33.64 |
| 39 | DBWH 221 | NA | IIWBR | India | Hexaploid | 0.48 | 5.58 | 3.46 | 12.50 | 70.33 | 30.30 |
| 40 | DL 153‐2 (KUNDAN) | NA | CCS HAU | India | Hexaploid | 0.81 | 6.13 | 3.80 | 15.30 | 67.60 | 45.70 |
| 41 | DL 784‐3 (VAISHALI) | NA | CCS HAU | India | Hexaploid | 0.73 | 5.25 | 3.25 | 17.90 | 62.30 | 22.60 |
| 42 | DL 788‐2 (VIDISHA) | NA | CCS HAU | India | Hexaploid | 0.72 | 5.80 | 3.59 | 16.30 | 64.60 | 33.00 |
| 43 | DL 803‐3 (KANCHAN) | NA | CCS HAU | India | Hexaploid | 0.87 | 5.04 | 3.13 | 15.07 | 66.80 | 37.60 |
| 44 | DPW 621-50 | NA | CCS HAU | India | Hexaploid | 0.84 | 6.09 | 3.78 | 10.70 | 70.20 | 47.60 |
| 45 | DURGAPURA 65 | NA | CCS HAU | India | Hexaploid | 0.77 | 5.96 | 3.69 | 14.97 | 67.43 | 35.00 |
| 46 | DWR 16 (KEERTHI) | NA | CCS HAU | India | Hexaploid | 0.63 | 6.05 | 3.75 | 16.80 | 66.20 | 33.50 |
| 47 | DWR 195 (ANURADHA) | NA | CCS HAU | India | Hexaploid | 0.56 | 6.44 | 3.99 | 17.10 | 65.50 | 31.50 |
| 48 | DWR 39 (PRAGATI) | NA | CCS HAU | India | Hexaploid | 0.85 | 5.25 | 3.25 | 13.60 | 69.60 | 29.10 |
| 49 | DWR‐162 | NA | CCS HAU | India | Hexaploid | 0.64 | 5.97 | 3.70 | 15.90 | 67.40 | 38.00 |
| 50 | FEDERATION | NA | CCS HAU | India | Hexaploid | 0.84 | 6.24 | 3.87 | 14.17 | 65.90 | 25.70 |
| 51 | GABO | NA | CCS HAU | India | Hexaploid | 0.76 | 5.36 | 3.33 | 15.13 | 66.60 | 22.50 |
| 52 | GW 10 | NA | CCS HAU | India | Hexaploid | 1.20 | 6.09 | 3.78 | 16.90 | 63.80 | 28.80 |
| 53 | GW 120 | NA | CCS HAU | India | Hexaploid | 0.76 | 5.49 | 3.40 | 15.40 | 65.53 | 28.50 |
| 54 | GW 173 | NA | CCS HAU | India | Hexaploid | 0.79 | 5.47 | 3.39 | 15.63 | 67.70 | 26.80 |
| 55 | GW 190 | NA | CCS HAU | India | Hexaploid | 0.71 | 6.64 | 4.12 | 15.30 | 65.90 | 35.60 |
| 56 | GW 273 | NA | CCS HAU | India | Hexaploid | 0.87 | 5.35 | 3.32 | 15.60 | 65.80 | 23.30 |
| 57 | GW 322 | NA | CCS HAU | India | Hexaploid | 0.81 | 5.65 | 3.50 | 15.70 | 66.83 | 27.60 |
| 58 | GW 40 | NA | CCS HAU | India | Hexaploid | 0.81 | 5.54 | 3.43 | 15.90 | 66.90 | 25.50 |
| 59 | GW 496 | NA | CCS HAU | India | Hexaploid | 0.87 | 5.82 | 3.61 | 12.40 | 72.07 | 37.50 |
| 60 | GW 503 | NA | CCS HAU | India | Hexaploid | 0.67 | 4.84 | 3.00 | 13.20 | 70.40 | 22.80 |
| 61 | GW 89 | NA | CCS HAU | India | Hexaploid | 0.79 | 5.33 | 3.30 | 13.17 | 69.00 | 30.20 |
| 62 | HB 208 | NA | CCS HAU | India | Hexaploid | 0.78 | 5.44 | 3.38 | 12.40 | 68.70 | 42.00 |
| 63 | HBLY 2015-07 | NA | CCS HAU | India | Hexaploid | 0.76 | 5.35 | 3.32 | 12.10 | 70.93 | 37.04 |
| 64 | HD 1925 (SHERA) | NA | CCS HAU | India | Hexaploid | 0.81 | 4.26 | 2.64 | 12.80 | 70.40 | 46.30 |
| 65 | HD 1941 (HIRA) | NA | CCS HAU | India | Hexaploid | 0.85 | 5.39 | 3.34 | 15.60 | 65.77 | 31.10 |
| 66 | HD 1949 (MOTI) | NA | CCS HAU | India | Hexaploid | 0.75 | 6.14 | 3.81 | 14.70 | 67.00 | 25.70 |
| 67 | HD 1981 (PRATAP) | NA | CCS HAU | India | Hexaploid | 0.78 | 5.20 | 3.23 | 14.80 | 67.67 | 22.80 |
| 68 | HD 1982 (JANAK) | NA | CCS HAU | India | Hexaploid | 0.81 | 5.36 | 3.33 | 15.20 | 68.07 | 26.80 |
| 69 | HD 2009 | NA | CCS HAU | India | Hexaploid | 0.79 | 6.35 | 3.93 | 10.40 | 71.40 | 38.54 |
| 70 | HD 2135 (NILGIRI) | NA | CCS HAU | India | Hexaploid | 0.83 | 5.52 | 3.42 | 14.90 | 67.40 | 27.70 |
| 71 | HD 2177 | NA | CCS HAU | India | Hexaploid | 0.84 | 5.79 | 3.59 | 15.20 | 67.70 | 31.80 |
| 72 | HD 2189 | NA | CCS HAU | India | Hexaploid | 0.79 | 4.84 | 3.00 | 12.70 | 71.10 | 42.20 |
| 73 | HD 2236 (KSHIPRA) | NA | CCS HAU | India | Hexaploid | 0.94 | 4.94 | 3.06 | 13.30 | 69.10 | 33.90 |
| 74 | HD 2270 | NA | CCS HAU | India | Hexaploid | 0.82 | 5.15 | 3.19 | 10.80 | 72.30 | 34.80 |
| 75 | HD 2278 (PARVATI) | NA | CCS HAU | India | Hexaploid | 0.75 | 5.47 | 3.39 | 11.50 | 72.50 | 31.20 |
| 76 | HD 2281 | NA | CCS HAU | India | Hexaploid | 0.70 | 5.96 | 3.69 | 12.73 | 70.00 | 33.20 |
| 77 | HD 2285 (GOBIND) | NA | CCS HAU | India | Hexaploid | 0.84 | 4.74 | 2.94 | 12.17 | 71.50 | 35.60 |
| 78 | HD 2307 | NA | CCS HAU | India | Hexaploid | 0.82 | 4.91 | 3.04 | 11.60 | 71.87 | 34.50 |
| 79 | HD 2327 | NA | CCS HAU | India | Hexaploid | 0.72 | 4.95 | 3.07 | 10.90 | 73.20 | 38.40 |
| 80 | HD 2329 | NA | CCS HAU | India | Hexaploid | 0.98 | 5.71 | 3.54 | 13.60 | 69.90 | 43.70 |
| 81 | HD 2380 | NA | CCS HAU | India | Hexaploid | 0.85 | 5.40 | 3.35 | 10.10 | 73.40 | 42.90 |
| 82 | HD 2385 | NA | CCS HAU | India | Hexaploid | 0.74 | 5.93 | 3.68 | 11.47 | 72.40 | 32.90 |
| 83 | HD 2402 | NA | CCS HAU | India | Hexaploid | 1.05 | 6.12 | 3.79 | 10.97 | 72.10 | 42.60 |
| 84 | HD 2501 | NA | CCS HAU | India | Hexaploid | 0.81 | 5.30 | 3.28 | 13.40 | 70.53 | 31.50 |
| 85 | HD 2643 (GANGA) | NA | CCS HAU | India | Hexaploid | 0.70 | 6.19 | 3.84 | 15.50 | 66.90 | 33.50 |
| 86 | HD 2687 | NA | CCS HAU | India | Hexaploid | 0.75 | 6.13 | 3.80 | 10.20 | 70.20 | 42.20 |
| 87 | HD 2733 (VMS) | NA | CCS HAU | India | Hexaploid | 0.56 | 6.30 | 3.91 | 14.50 | 67.00 | 27.70 |
| 88 | HD 2781 (ADITYA) | NA | CCS HAU | India | Hexaploid | 0.66 | 6.37 | 3.95 | 13.83 | 69.17 | 38.10 |
| 89 | HD 2824 | NA | CCS HAU | India | Hexaploid | 0.64 | 6.23 | 3.86 | 14.53 | 68.80 | 32.00 |
| 90 | HD 2833 (TRIPTI) | NA | CCS HAU | India | Hexaploid | 0.87 | 5.39 | 3.34 | 13.97 | 68.50 | 27.60 |
| 91 | HD 2851 | NA | CCS HAU | India | Hexaploid | 1.04 | 7.24 | 4.49 | 11.50 | 71.20 | 47.90 |
| 92 | HD 2864 (URJA) | NA | CCS HAU | India | Hexaploid | 0.80 | 4.55 | 2.82 | 14.10 | 66.60 | 27.60 |
| 93 | HD 2894 | NA | CCS HAU | India | Hexaploid | 0.92 | 6.52 | 4.04 | 11.83 | 70.90 | 44.40 |
| 94 | HD 2967 | NA | CCS HAU | India | Hexaploid | 0.80 | 7.10 | 4.40 | 10.60 | 70.80 | 44.10 |
| 95 | HD 2968 | NA | CCS HAU | India | Hexaploid | 0.75 | 6.56 | 4.07 | 10.73 | 70.50 | 50.90 |
| 96 | HD 2987 | NA | CCS HAU | India | Hexaploid | 0.75 | 7.34 | 4.55 | 12.00 | 68.30 | 43.84 |
| 97 | HD 3011 | NA | CCS HAU | India | Hexaploid | 0.96 | 6.73 | 4.17 | 10.80 | 70.40 | 44.24 |
| 98 | HD 3043 | NA | CCS HAU | India | Hexaploid | 0.84 | 6.36 | 3.94 | 10.93 | 69.80 | 42.34 |
| 99 | HD 3059 | NA | CCS HAU | India | Hexaploid | 0.79 | 6.94 | 4.30 | 10.50 | 70.50 | 47.70 |
| 100 | HD 3086 | NA | CCS HAU | India | Hexaploid | 0.90 | 7.04 | 4.37 | 10.10 | 71.77 | 45.40 |
| 101 | HD 3182 | NA | CCS HAU | India | Hexaploid | 0.80 | 5.79 | 3.59 | 10.67 | 72.20 | 49.70 |
| 102 | HD 3219 | NA | CCS HAU | India | Hexaploid | 0.84 | 7.49 | 4.64 | 11.10 | 69.57 | 45.60 |
| 103 | HD 3226 | NA | CCS HAU | India | Hexaploid | 0.82 | 6.08 | 3.77 | 11.83 | 70.53 | 39.54 |
| 104 | HD‐2204 | NA | CCS HAU | India | Hexaploid | 0.81 | 4.92 | 3.05 | 13.23 | 69.90 | 30.90 |
| 105 | HDR 77 | NA | CCS HAU | India | Hexaploid | 0.74 | 4.72 | 2.93 | 13.20 | 69.80 | 24.70 |
| 106 | HI 1077 (MANGLA) | NA | CCS HAU | India | Hexaploid | 0.63 | 5.49 | 3.40 | 12.47 | 70.27 | 32.10 |
| 107 | HI 1418 (NAVEEN CHANDOUSI) | NA | CCS HAU | India | Hexaploid | 0.81 | 5.77 | 3.58 | 12.97 | 68.90 | 28.90 |
| 108 | HI 1454 (ABHA) | NA | CCS HAU | India | Hexaploid | 0.79 | 5.85 | 3.63 | 13.23 | 68.47 | 37.10 |
| 109 | HI 1500 (AMRITA) | NA | CCS HAU | India | Hexaploid | 0.69 | 6.84 | 4.24 | 13.27 | 68.80 | 40.20 |
| 110 | HI 1621 | NA | CCS HAU | India | Hexaploid | 0.78 | 5.57 | 3.45 | 11.40 | 69.33 | 47.20 |
| 111 | HI 1625 | NA | CCS HAU | India | Hexaploid | 0.80 | 6.69 | 4.15 | 11.50 | 70.03 | 44.60 |
| 112 | HI 1628 | NA | CCS HAU | India | Hexaploid | 0.82 | 5.72 | 3.55 | 11.83 | 70.33 | 42.30 |
| 113 | HI 617 (SUJATA) | NA | CCS HAU | India | Hexaploid | 0.96 | 6.85 | 4.25 | 10.53 | 71.97 | 49.50 |
| 114 | HI 784 (SWATI) | NA | CCS HAU | India | Hexaploid | 0.69 | 4.54 | 2.82 | 10.20 | 71.40 | 32.40 |
| 115 | HI 977 | NA | CCS HAU | India | Hexaploid | 0.71 | 5.64 | 3.50 | 12.77 | 67.90 | 33.00 |
| 116 | HP 1102 | NA | CCS HAU | India | Hexaploid | 0.75 | 5.46 | 3.38 | 11.27 | 70.00 | 36.60 |
| 117 | HP 1493 | NA | CCS HAU | India | Hexaploid | 0.99 | 5.18 | 3.21 | 12.77 | 70.70 | 37.90 |
| 118 | HP 1633 (SONALI) | NA | CCS HAU | India | Hexaploid | 0.90 | 4.50 | 2.79 | 11.70 | 71.90 | 21.80 |
| 119 | HP 1731 (RAJ LAKSHMI) | NA | CCS HAU | India | Hexaploid | 0.91 | 4.88 | 3.03 | 12.60 | 69.33 | 31.90 |
| 120 | HP 1744 (RAJESHWARI) | NA | CCS HAU | India | Hexaploid | 0.85 | 5.57 | 3.45 | 10.90 | 72.23 | 45.10 |
| 121 | HP 1761 (JAGDISH) | NA | CCS HAU | India | Hexaploid | 0.89 | 5.32 | 3.30 | 12.57 | 70.00 | 29.60 |
| 122 | HPBW 01 | NA | CCS HAU | India | Hexaploid | 0.96 | 6.16 | 3.82 | 11.80 | 70.13 | 45.30 |
| 123 | HPBW 07 | NA | CCS HAU | India | Hexaploid | 1.06 | 5.97 | 3.70 | 13.20 | 69.80 | 36.20 |
| 124 | HPBW 09 | NA | CCS HAU | India | Hexaploid | 0.74 | 6.33 | 3.93 | 13.30 | 68.47 | 39.20 |
| 125 | HPW 184 (CHANDRIKA) | NA | CCS HAU | India | Hexaploid | 0.66 | 6.31 | 3.91 | 13.77 | 69.53 | 27.60 |
| 126 | HPW 42 (ARADHANA) | NA | CCS HAU | India | Hexaploid | 0.61 | 5.80 | 3.59 | 14.30 | 69.10 | 24.10 |
| 127 | HPW 89 (SURABHI) | NA | CCS HAU | India | Hexaploid | 0.66 | 5.48 | 3.40 | 13.37 | 69.53 | 35.60 |
| 128 | HPW‐147 | NA | CCS HAU | India | Hexaploid | 0.70 | 5.44 | 3.38 | 14.50 | 68.07 | 40.80 |
| 129 | HS 1097‐17 (GIRIJA) | NA | CCS HAU | India | Hexaploid | 0.85 | 5.42 | 3.36 | 12.60 | 71.77 | 40.50 |
| 130 | HS 1138‐6‐4 (SHAILJA) | NA | CCS HAU | India | Hexaploid | 0.80 | 4.83 | 2.99 | 9.43 | 73.73 | 36.10 |
| 131 | HS 207 | NA | CCS HAU | India | Hexaploid | 0.86 | 5.80 | 3.59 | 10.50 | 68.40 | 45.90 |
| 132 | HS 240 | NA | CCS HAU | India | Hexaploid | 0.86 | 5.95 | 3.69 | 12.83 | 70.07 | 27.40 |
| 133 | HS 277 | NA | CCS HAU | India | Hexaploid | 0.93 | 5.40 | 3.35 | 12.20 | 69.20 | 34.30 |
| 134 | HS 295 | NA | CCS HAU | India | Hexaploid | 0.61 | 4.86 | 3.01 | 11.63 | 72.20 | 32.10 |
| 135 | HS 365 | NA | CCS HAU | India | Hexaploid | 0.95 | 5.31 | 3.29 | 12.33 | 70.10 | 26.20 |
| 136 | HS 375 (HIMGIRI) | NA | CCS HAU | India | Hexaploid | 0.74 | 5.43 | 3.37 | 13.40 | 70.00 | 32.20 |
| 137 | HS 420 (SHIVALIK) | NA | CCS HAU | India | Hexaploid | 0.86 | 5.27 | 3.27 | 11.13 | 71.40 | 41.90 |
| 138 | HS 86 | NA | CCS HAU | India | Hexaploid | 0.75 | 5.42 | 3.36 | 10.90 | 72.57 | 28.80 |
| 139 | HUW 12 (MALVIYA 12) | NA | CCS HAU | India | Hexaploid | 1.01 | 6.67 | 4.13 | 12.80 | 70.30 | 38.10 |
| 140 | HUW 206 (MALVIYA 206) | NA | CCS HAU | India | Hexaploid | 0.58 | 6.08 | 3.77 | 15.90 | 67.70 | 30.50 |
| 141 | HUW 213 | NA | CCS HAU | India | Hexaploid | 0.74 | 5.19 | 3.22 | 15.27 | 65.40 | 31.70 |
| 142 | HUW 234 | NA | CCS HAU | India | Hexaploid | 0.82 | 5.14 | 3.18 | 11.40 | 72.90 | 45.64 |
| 143 | HUW 37 (MALVIYA 37) | NA | CCS HAU | India | Hexaploid | 1.01 | 5.03 | 3.12 | 13.63 | 64.40 | 21.50 |
| 144 | HUW 540 | NA | CCS HAU | India | Hexaploid | 0.81 | 6.86 | 4.25 | 12.00 | 69.80 | 49.10 |
| 145 | HUW 55 (MALVIYA 55) | NA | CCS HAU | India | Hexaploid | 0.65 | 4.77 | 2.96 | 13.00 | 70.50 | 21.00 |
| 146 | HUW 615 | NA | CCS HAU | India | Hexaploid | 0.82 | 5.47 | 3.39 | 11.80 | 71.10 | 47.10 |
| 147 | HUW 702 | NA | CCS HAU | India | Hexaploid | 0.79 | 6.65 | 4.13 | 12.70 | 68.50 | 40.44 |
| 148 | HUW 704 | NA | CCS HAU | India | Hexaploid | 0.79 | 5.63 | 3.49 | 12.30 | 71.00 | 43.40 |
| 149 | HW 1085 (BHAWANI) | NA | CCS HAU | India | Hexaploid | 0.92 | 6.23 | 3.86 | 13.03 | 70.00 | 37.10 |
| 150 | HW 2004 (AMAR) | NA | CCS HAU | India | Hexaploid | 0.87 | 6.24 | 3.87 | 13.10 | 69.10 | 44.30 |
| 151 | HW 2045 (KAUSHAMBI) | NA | CCS HAU | India | Hexaploid | 1.07 | 5.92 | 3.67 | 12.33 | 70.07 | 30.80 |
| 152 | HW 517 | NA | CCS HAU | India | Hexaploid | 0.80 | 5.40 | 3.35 | 13.80 | 69.70 | 33.00 |
| 153 | HW 657 | NA | CCS HAU | India | Hexaploid | 0.87 | 5.08 | 3.15 | 12.90 | 68.40 | 30.70 |
| 154 | HW 741 | NA | CCS HAU | India | Hexaploid | 0.91 | 4.52 | 2.80 | 12.70 | 69.90 | 35.80 |
| 155 | HY 12 | NA | CCS HAU | India | Hexaploid | 0.68 | 5.93 | 3.68 | 14.60 | 68.60 | 39.90 |
| 156 | HY 5 | NA | CCS HAU | India | Hexaploid | 0.84 | 5.72 | 3.55 | 15.00 | 66.63 | 41.80 |
| 157 | HYB 11 | NA | CCS HAU | India | Hexaploid | 0.70 | 6.72 | 4.17 | 12.50 | 68.80 | 32.70 |
| 158 | HYB 277 | NA | CCS HAU | India | Hexaploid | 0.99 | 5.51 | 3.42 | 12.93 | 69.90 | 32.50 |
| 159 | HYB 633 | NA | CCS HAU | India | Hexaploid | 0.83 | 4.87 | 3.02 | 13.63 | 69.30 | 32.00 |
| 160 | HYB 65 | NA | CCS HAU | India | Hexaploid | 0.77 | 4.85 | 3.01 | 13.73 | 68.70 | 21.90 |
| 161 | IWP 72 | NA | CCS HAU | India | Hexaploid | 0.94 | 6.31 | 3.91 | 11.33 | 71.90 | 33.70 |
| 162 | J‐1‐7 | NA | CCS HAU | India | Hexaploid | 0.84 | 5.26 | 3.26 | 10.10 | 71.70 | 32.40 |
| 163 | J‐24 | NA | CCS HAU | India | Hexaploid | 0.86 | 6.25 | 3.88 | 10.10 | 71.47 | 38.30 |
| 164 | J‐405 | NA | CCS HAU | India | Hexaploid | 0.90 | 5.11 | 3.17 | 11.17 | 70.43 | 36.00 |
| 165 | JAUW 635 | NA | CCS HAU | India | Hexaploid | 0.86 | 5.75 | 3.57 | 10.23 | 71.20 | 43.70 |
| 166 | JWS 17 (SWAPNIL) | NA | CCS HAU | India | Hexaploid | 0.86 | 5.04 | 3.13 | 11.30 | 71.90 | 34.60 |
| 167 | K 4117 | NA | CCS HAU | India | Hexaploid | 0.92 | 4.86 | 3.01 | 12.30 | 70.43 | 51.50 |
| 168 | K 53 | NA | CCS HAU | India | Hexaploid | 0.92 | 5.74 | 3.56 | 11.50 | 69.13 | 44.00 |
| 169 | K 65 | NA | CCS HAU | India | Hexaploid | 0.85 | 5.01 | 3.11 | 10.50 | 72.80 | 49.70 |
| 170 | K 7410 (SHEKHAR) | NA | CCS HAU | India | Hexaploid | 0.90 | 5.23 | 3.24 | 13.40 | 67.00 | 21.00 |
| 171 | K 78 | NA | CCS HAU | India | Hexaploid | 0.82 | 5.72 | 3.55 | 13.80 | 68.50 | 34.10 |
| 172 | K 7903 (HALNA) | NA | CCS HAU | India | Hexaploid | 0.92 | 5.63 | 3.49 | 14.30 | 66.40 | 16.50 |
| 173 | K 8020 (TRIVENI) | NA | CCS HAU | India | Hexaploid | 0.86 | 4.09 | 2.53 | 15.00 | 67.30 | 29.90 |
| 174 | K 8027 (MAGHAR) | NA | CCS HAU | India | Hexaploid | 0.89 | 5.68 | 3.52 | 14.07 | 69.67 | 44.50 |
| 175 | K 816 | NA | CCS HAU | India | Hexaploid | 0.77 | 6.33 | 3.93 | 13.80 | 67.87 | 21.00 |
| 176 | K 8434 (PRASAD) | NA | CCS HAU | India | Hexaploid | 0.81 | 5.04 | 3.13 | 14.70 | 69.00 | 31.50 |
| 177 | K 88 (K8804) | NA | CCS HAU | India | Hexaploid | 0.73 | 5.75 | 3.57 | 15.63 | 65.83 | 35.50 |
| 178 | K 8962 (INDRA) | NA | CCS HAU | India | Hexaploid | 0.99 | 5.55 | 3.44 | 12.90 | 70.00 | 36.30 |
| 179 | K 9006 (UJIAYR) | NA | CCS HAU | India | Hexaploid | 1.00 | 4.76 | 2.95 | 13.00 | 71.40 | 31.50 |
| 180 | K 9162 | NA | CCS HAU | India | Hexaploid | 0.83 | 5.74 | 3.56 | 14.07 | 68.80 | 47.20 |
| 181 | K 9351 (MANDAKINI) | NA | CCS HAU | India | Hexaploid | 0.92 | 5.18 | 3.21 | 12.70 | 70.33 | 33.10 |
| 182 | K 9423 (UNNAT HALNA) | NA | CCS HAU | India | Hexaploid | 0.89 | 4.76 | 2.95 | 15.70 | 67.50 | 34.60 |
| 183 | K 9533 (NAINA) | NA | CCS HAU | India | Hexaploid | 0.81 | 4.68 | 2.90 | 11.10 | 72.10 | 38.00 |
| 184 | K 9644 (ATAL) | NA | CCS HAU | India | Hexaploid | 1.17 | 5.19 | 3.22 | 12.70 | 70.60 | 36.60 |
| 185 | KALYAN SONA | NA | CCS HAU | India | Hexaploid | 1.03 | 5.98 | 3.71 | 11.00 | 71.30 | 47.60 |
| 186 | KENPHAD 25 | NA | CCS HAU | India | Hexaploid | 0.79 | 4.60 | 2.85 | 12.87 | 68.20 | 23.00 |
| 187 | KHARCHIA 65 | NA | CCS HAU | India | Hexaploid | 0.93 | 5.60 | 3.47 | 13.23 | 69.10 | 27.40 |
| 188 | KRL 1‐4 | NA | CCS HAU | India | Hexaploid | 0.95 | 5.12 | 3.18 | 10.60 | 72.90 | 42.00 |
| 189 | KRL 19 | NA | CCS HAU | India | Hexaploid | 0.82 | 4.65 | 2.88 | 11.40 | 72.57 | 34.00 |
| 190 | KRL 730 | NA | CCS HAU | India | Hexaploid | 0.84 | 5.52 | 3.42 | 9.50 | 71.20 | 42.30 |
| 191 | KSML 3 | NA | CCS HAU | India | Hexaploid | 0.80 | 4.57 | 2.83 | 11.40 | 72.43 | 37.60 |
| 192 | LAL BAHADUR | NA | CCS HAU | India | Hexaploid | 0.90 | 4.53 | 2.81 | 13.70 | 70.53 | 40.10 |
| 193 | LOK 1 | NA | CCS HAU | India | Hexaploid | 0.97 | 5.96 | 3.69 | 13.40 | 70.93 | 45.20 |
| 194 | LOK 54 | NA | CCS HAU | India | Hexaploid | 0.90 | 6.48 | 4.02 | 11.70 | 70.50 | 48.20 |
| 195 | MACS 2496 | NA | CCS HAU | India | Hexaploid | 0.67 | 5.83 | 3.62 | 11.10 | 70.53 | 35.60 |
| 196 | MLKS 11 | NA | CCS HAU | India | Hexaploid | 0.82 | 5.20 | 3.23 | 11.37 | 69.77 | 28.70 |
| 197 | MONDHYA 3‐2 | NA | CCS HAU | India | Hexaploid | 0.94 | 5.97 | 3.70 | 10.30 | 73.10 | 39.10 |
| 198 | NARMADA 112 | NA | CCS HAU | India | Hexaploid | 0.88 | 6.44 | 3.99 | 11.60 | 71.07 | 44.10 |
| 199 | NARMADA 195 | NA | CCS HAU | India | Hexaploid | 0.86 | 6.07 | 3.76 | 13.57 | 69.87 | 47.10 |
| 200 | NARMADA 4 | NA | CCS HAU | India | Hexaploid | 0.79 | 5.48 | 3.40 | 13.73 | 69.23 | 40.60 |
| 201 | NBRL 2015-07 | NA | CCS HAU | India | Hexaploid | 0.97 | 5.46 | 3.38 | 11.60 | 69.00 | 49.60 |
| 202 | NI 179 | NA | CCS HAU | India | Hexaploid | 0.75 | 4.99 | 3.09 | 12.17 | 69.67 | 33.60 |
| 203 | NI 345 | NA | CCS HAU | India | Hexaploid | 0.53 | 6.21 | 3.85 | 11.93 | 70.47 | 35.30 |
| 204 | NI 5439 | NA | CCS HAU | India | Hexaploid | 0.81 | 5.09 | 3.16 | 11.00 | 71.60 | 29.20 |
| 205 | NI 5643 | NA | CCS HAU | India | Hexaploid | 0.75 | 6.05 | 3.75 | 11.40 | 71.03 | 33.60 |
| 206 | NIAW 301 (TRIMBAK) | NA | CCS HAU | India | Hexaploid | 0.65 | 4.86 | 3.01 | 11.20 | 71.40 | 32.40 |
| 207 | NIAW 3170 | NA | CCS HAU | India | Hexaploid | 0.81 | 5.39 | 3.34 | 10.70 | 71.40 | 41.40 |
| 208 | NIAW‐34 | NA | CCS HAU | India | Hexaploid | 0.62 | 5.58 | 3.46 | 11.20 | 71.97 | 42.00 |
| 209 | NP 100 | NA | CCS HAU | India | Hexaploid | 0.37 | 5.33 | 3.30 | 12.20 | 72.70 | 31.00 |
| 210 | NP 101 | NA | CCS HAU | India | Hexaploid | 0.31 | 5.26 | 3.26 | 12.57 | 70.90 | 42.60 |
| 211 | NP 111 | NA | CCS HAU | India | Hexaploid | 0.44 | 5.46 | 3.38 | 13.30 | 69.90 | 37.80 |
| 212 | NP 114 | NA | CCS HAU | India | Hexaploid | 0.33 | 5.17 | 3.21 | 14.90 | 71.37 | 35.10 |
| 213 | NP 12 | NA | CCS HAU | India | Hexaploid | 0.45 | 5.98 | 3.71 | 17.20 | 66.40 | 26.30 |
| 214 | NP 120 | NA | CCS HAU | India | Hexaploid | 0.88 | 5.47 | 3.39 | 15.37 | 68.10 | 35.30 |
| 215 | NP 165 | NA | CCS HAU | India | Hexaploid | 0.61 | 5.10 | 3.16 | 14.90 | 69.63 | 38.00 |
| 216 | NP 4 | NA | CCS HAU | India | Hexaploid | 0.46 | 5.33 | 3.30 | 13.67 | 69.40 | 45.00 |
| 217 | NP 52 | NA | CCS HAU | India | Hexaploid | 0.90 | 5.85 | 3.63 | 12.20 | 71.47 | 38.00 |
| 218 | NP 710 | NA | CCS HAU | India | Hexaploid | 0.72 | 4.55 | 2.82 | 12.70 | 70.57 | 49.80 |
| 219 | NP 715 | NA | CCS HAU | India | Hexaploid | 0.88 | 5.00 | 3.10 | 14.60 | 69.80 | 38.80 |
| 220 | NP 718 | NA | CCS HAU | India | Hexaploid | 0.88 | 5.04 | 3.13 | 15.30 | 69.20 | 36.60 |
| 221 | NP 721 | NA | CCS HAU | India | Hexaploid | 0.76 | 4.87 | 3.02 | 14.20 | 69.83 | 38.50 |
| 222 | NP 745 | NA | CCS HAU | India | Hexaploid | 0.96 | 5.84 | 3.62 | 11.20 | 72.60 | 32.70 |
| 223 | NP 760 | NA | CCS HAU | India | Hexaploid | 0.69 | 4.75 | 2.94 | 11.17 | 71.90 | 16.80 |
| 224 | NP 761 | NA | CCS HAU | India | Hexaploid | 0.77 | 5.60 | 3.47 | 11.30 | 72.40 | 46.90 |
| 225 | NP 770 | NA | CCS HAU | India | Hexaploid | 0.90 | 6.37 | 3.95 | 12.67 | 70.50 | 36.90 |
| 226 | NP 771 | NA | CCS HAU | India | Hexaploid | 0.70 | 6.16 | 3.82 | 13.00 | 71.40 | 37.10 |
| 227 | NP 792 | NA | CCS HAU | India | Hexaploid | 0.68 | 6.01 | 3.73 | 13.37 | 71.20 | 35.40 |
| 228 | NP 799 | NA | CCS HAU | India | Hexaploid | 0.74 | 6.00 | 3.72 | 14.60 | 70.00 | 39.00 |
| 229 | NP 809 | NA | CCS HAU | India | Hexaploid | 0.69 | 6.29 | 3.90 | 13.43 | 70.63 | 37.40 |
| 230 | NP 818 | NA | CCS HAU | India | Hexaploid | 0.75 | 6.36 | 3.94 | 13.80 | 69.53 | 36.10 |
| 231 | NP 823 | NA | CCS HAU | India | Hexaploid | 0.76 | 6.64 | 4.12 | 11.80 | 70.80 | 39.90 |
| 232 | NP 824 | NA | CCS HAU | India | Hexaploid | 0.71 | 6.09 | 3.78 | 11.87 | 72.43 | 31.70 |
| 233 | NP 825 | NA | CCS HAU | India | Hexaploid | 0.88 | 6.38 | 3.96 | 11.70 | 71.20 | 35.10 |
| 234 | NP 830 | NA | CCS HAU | India | Hexaploid | 0.74 | 5.87 | 3.64 | 14.10 | 69.67 | 33.00 |
| 235 | NP 832 | NA | CCS HAU | India | Hexaploid | 0.88 | 5.72 | 3.55 | 13.60 | 70.40 | 37.70 |
| 236 | NP 836 | NA | CCS HAU | India | Hexaploid | 0.95 | 5.30 | 3.28 | 13.17 | 69.97 | 38.80 |
| 237 | NP 839 | NA | CCS HAU | India | Hexaploid | 0.82 | 5.14 | 3.18 | 13.90 | 67.50 | 46.80 |
| 238 | NP 852 | NA | CCS HAU | India | Hexaploid | 0.71 | 4.71 | 2.92 | 12.80 | 66.50 | 18.00 |
| 239 | NP 884 | NA | CCS HAU | India | Hexaploid | 0.80 | 5.00 | 3.10 | 14.60 | 69.80 | 30.90 |
| 240 | NP 890 | NA | CCS HAU | India | Hexaploid | 0.74 | 5.99 | 3.71 | 16.10 | 68.50 | 39.70 |
| 241 | NW 1067 | NA | CCS HAU | India | Hexaploid | 0.69 | 5.73 | 3.55 | 12.77 | 69.23 | 38.70 |
| 242 | NW 1076 | NA | CCS HAU | India | Hexaploid | 0.75 | 5.64 | 3.50 | 11.60 | 71.50 | 34.20 |
| 243 | NW 2036 | NA | CCS HAU | India | Hexaploid | 0.68 | 5.51 | 3.42 | 14.03 | 68.70 | 31.30 |
| 244 | NW 2306 | NA | CCS HAU | India | Hexaploid | 0.91 | 6.60 | 4.09 | 13.80 | 66.83 | 53.20 |
| 245 | NW 7041 | NA | CCS HAU | India | Hexaploid | 0.78 | 6.16 | 3.82 | 12.67 | 68.30 | 48.40 |
| 246 | NW 7049 | NA | CCS HAU | India | Hexaploid | 0.75 | 5.66 | 3.51 | 12.57 | 71.10 | 46.00 |
| 247 | NW 707 | NA | CCS HAU | India | Hexaploid | 0.96 | 6.20 | 3.84 | 13.40 | 70.10 | 52.30 |
| 248 | P 11638 | NA | CCS HAU | India | Hexaploid | 0.84 | 6.84 | 4.24 | 11.10 | 71.50 | 52.10 |
| 249 | PBC TYPE II (TYPE‐II) | NA | CCS HAU | India | Hexaploid | 0.79 | 5.74 | 3.56 | 11.97 | 71.80 | 41.50 |
| 250 | PBN 142 (KAILASH) | NA | CCS HAU | India | Hexaploid | 0.66 | 4.88 | 3.03 | 13.30 | 68.80 | 28.80 |
| 251 | PBN 51 | NA | CCS HAU | India | Hexaploid | 0.85 | 5.06 | 3.13 | 11.77 | 70.83 | 34.20 |
| 252 | PBW 12 | NA | CCS HAU | India | Hexaploid | 0.79 | 5.22 | 3.23 | 12.90 | 68.20 | 37.40 |
| 253 | PBW 120 | NA | CCS HAU | India | Hexaploid | 0.73 | 5.11 | 3.17 | 9.20 | 73.80 | 32.50 |
| 254 | PBW 123 | NA | CCS HAU | India | Hexaploid | 1.03 | 6.16 | 3.82 | 14.40 | 67.20 | 51.14 |
| 255 | PBW 138 | NA | CCS HAU | India | Hexaploid | 0.84 | 5.24 | 3.25 | 9.60 | 73.70 | 49.60 |
| 256 | PBW 154 | NA | CCS HAU | India | Hexaploid | 0.82 | 5.06 | 3.13 | 11.10 | 73.30 | 37.70 |
| 257 | PBW 158 | NA | CCS HAU | India | Hexaploid | 0.97 | 5.82 | 3.61 | 12.07 | 69.80 | 44.64 |
| 258 | PBW 163 | NA | CCS HAU | India | Hexaploid | 1.10 | 6.85 | 4.25 | 13.60 | 68.30 | 38.64 |
| 259 | PBW 165 | NA | CCS HAU | India | Hexaploid | 0.92 | 5.25 | 3.25 | 14.40 | 67.60 | 52.44 |
| 260 | PBW 175 | NA | CCS HAU | India | Hexaploid | 1.11 | 7.54 | 4.68 | 11.80 | 72.03 | 46.64 |
| 261 | PBW 226 | NA | CCS HAU | India | Hexaploid | 0.75 | 6.27 | 3.88 | 15.63 | 66.60 | 24.80 |
| 262 | PBW 343 | NA | CCS HAU | India | Hexaploid | 0.61 | 6.11 | 3.79 | 13.93 | 68.90 | 28.80 |
| 263 | PBW 373 | NA | CCS HAU | India | Hexaploid | 0.74 | 6.16 | 3.82 | 13.10 | 67.90 | 27.50 |
| 264 | PBW 396 | NA | CCS HAU | India | Hexaploid | 1.07 | 5.74 | 3.56 | 12.47 | 68.97 | 30.80 |
| 265 | PBW 443 | NA | CCS HAU | India | Hexaploid | 0.71 | 5.34 | 3.31 | 14.17 | 69.03 | 25.20 |
| 266 | PBW 475 | NA | CCS HAU | India | Hexaploid | 0.98 | 7.95 | 4.93 | 11.30 | 71.80 | 34.65 |
| 267 | PBW 486 | NA | CCS HAU | India | Hexaploid | 0.86 | 5.48 | 3.40 | 12.00 | 69.80 | 38.24 |
| 268 | PBW 502 | NA | CCS HAU | India | Hexaploid | 0.99 | 6.68 | 4.14 | 12.17 | 69.60 | 48.30 |
| 269 | PBW 503 | NA | CCS HAU | India | Hexaploid | 0.95 | 7.35 | 4.56 | 12.80 | 69.90 | 39.14 |
| 270 | PBW 509 | NA | CCS HAU | India | Hexaploid | 0.73 | 6.14 | 3.81 | 13.00 | 70.60 | 38.80 |
| 271 | PBW 527 | NA | CCS HAU | India | Hexaploid | 1.03 | 5.82 | 3.61 | 13.90 | 66.50 | 43.64 |
| 272 | PBW 528 | NA | CCS HAU | India | Hexaploid | 0.92 | 4.45 | 2.76 | 14.10 | 68.30 | 40.44 |
| 273 | PBW 533 | NA | CCS HAU | India | Hexaploid | 1.06 | 7.45 | 4.62 | 11.60 | 68.60 | 39.40 |
| 274 | PBW 54 | NA | CCS HAU | India | Hexaploid | 0.70 | 5.24 | 3.25 | 12.13 | 72.10 | 36.70 |
| 275 | PBW 544 | NA | CCS HAU | India | Hexaploid | 0.93 | 4.45 | 2.76 | 14.03 | 66.63 | 42.64 |
| 276 | PBW 550 | NA | CCS HAU | India | Hexaploid | 0.77 | 6.49 | 4.03 | 14.17 | 68.37 | 40.64 |
| 277 | PBW 560 | NA | CCS HAU | India | Hexaploid | 0.92 | 5.48 | 3.40 | 10.60 | 71.60 | 50.00 |
| 278 | PBW 621 | NA | CCS HAU | India | Hexaploid | 0.84 | 5.36 | 3.33 | 11.60 | 70.30 | 50.50 |
| 279 | PBW 644 | NA | CCS HAU | India | Hexaploid | 0.83 | 4.91 | 3.04 | 10.50 | 71.13 | 44.14 |
| 280 | PBW 65 | NA | CCS HAU | India | Hexaploid | 0.83 | 5.41 | 3.35 | 10.90 | 72.00 | 43.50 |
| 281 | PBW 675 | NA | CCS HAU | India | Hexaploid | 0.94 | 5.02 | 3.11 | 12.27 | 69.70 | 35.54 |
| 282 | PBW 676 | NA | CCS HAU | India | Hexaploid | 0.88 | 5.48 | 3.40 | 13.20 | 69.77 | 45.50 |
| 283 | PBW 677 | NA | CCS HAU | India | Hexaploid | 1.01 | 4.45 | 2.76 | 13.40 | 69.13 | 34.74 |
| 284 | PBW 681 | NA | CCS HAU | India | Hexaploid | 0.94 | 5.48 | 3.40 | 13.00 | 71.30 | 38.74 |
| 285 | PBW 693 | NA | CCS HAU | India | Hexaploid | 1.01 | 5.71 | 3.54 | 11.30 | 71.20 | 41.34 |
| 286 | PBW 698 | NA | CCS HAU | India | Hexaploid | 0.90 | 5.71 | 3.54 | 11.90 | 70.23 | 41.10 |
| 287 | PBW 706 | NA | CCS HAU | India | Hexaploid | 1.01 | 4.68 | 2.90 | 12.13 | 70.10 | 53.80 |
| 288 | PBW 709 | NA | CCS HAU | India | Hexaploid | 0.93 | 5.48 | 3.40 | 14.57 | 67.30 | 50.50 |
| 289 | PBW 712 | NA | CCS HAU | India | Hexaploid | 0.95 | 5.02 | 3.11 | 13.60 | 68.77 | 46.80 |
| 290 | PBW 714 | NA | CCS HAU | India | Hexaploid | 0.77 | 5.71 | 3.54 | 13.50 | 68.20 | 40.74 |
| 291 | PBW 721 | NA | CCS HAU | India | Hexaploid | 0.99 | 4.79 | 2.97 | 13.27 | 68.70 | 38.44 |
| 292 | PBW 725 | NA | CCS HAU | India | Hexaploid | 0.83 | 5.93 | 3.68 | 13.83 | 67.40 | 45.34 |
| 293 | PBW 726 | NA | CCS HAU | India | Hexaploid | 0.87 | 4.91 | 3.04 | 11.90 | 70.10 | 52.44 |
| 294 | PBW 728 | NA | CCS HAU | India | Hexaploid | 1.04 | 5.25 | 3.25 | 11.90 | 71.27 | 41.54 |
| 295 | PBW 729 | NA | CCS HAU | India | Hexaploid | 0.94 | 5.02 | 3.11 | 12.60 | 71.20 | 45.50 |
| 296 | PBW 752 | NA | CCS HAU | India | Hexaploid | 0.77 | 5.02 | 3.11 | 12.27 | 70.00 | 50.20 |
| 297 | PBW 757 | NA | CCS HAU | India | Hexaploid | 0.75 | 4.57 | 2.83 | 12.40 | 70.90 | 49.40 |
| 298 | PBW 762 | NA | CCS HAU | India | Hexaploid | 0.76 | 5.36 | 3.33 | 11.30 | 71.50 | 44.84 |
| 299 | PBW 766 | NA | CCS HAU | India | Hexaploid | 1.16 | 6.93 | 4.30 | 12.00 | 70.00 | 45.94 |
| 300 | PBW 769 | NA | CCS HAU | India | Hexaploid | 1.07 | 5.59 | 3.47 | 10.47 | 70.50 | 48.70 |
| 301 | PBW 771 | NA | CCS HAU | India | Hexaploid | 0.91 | 4.57 | 2.83 | 12.57 | 70.53 | 45.04 |
| 302 | PBW 796 | NA | CCS HAU | India | Hexaploid | 0.95 | 4.45 | 2.76 | 11.60 | 69.87 | 51.60 |
| 303 | PBW 797 | NA | CCS HAU | India | Hexaploid | 0.76 | 4.45 | 2.76 | 12.17 | 69.90 | 44.64 |
| 304 | PBW 820 | NA | CCS HAU | India | Hexaploid | 0.82 | 5.82 | 3.61 | 12.40 | 69.40 | 37.54 |
| 305 | PBW 821 | NA | CCS HAU | India | Hexaploid | 0.91 | 6.73 | 4.17 | 11.97 | 71.10 | 47.40 |
| 306 | PBW 824 | NA | CCS HAU | India | Hexaploid | 0.93 | 4.68 | 2.90 | 11.70 | 71.20 | 45.04 |
| 307 | PBW 825 | NA | CCS HAU | India | Hexaploid | 1.14 | 6.37 | 3.95 | 12.27 | 68.17 | 46.54 |
| 308 | PHR 1022 | NA | CCS HAU | India | Hexaploid | 1.02 | 6.35 | 3.93 | 10.70 | 71.00 | 38.50 |
| 309 | PMW 621-50 | NA | CCS HAU | India | Hexaploid | 0.97 | 5.30 | 3.28 | 11.40 | 67.60 | 49.80 |
| 310 | Purple 3857 | NA | CCS HAU | India | Hexaploid | 0.78 | 4.66 | 2.89 | 10.40 | 66.17 | 44.10 |
| 311 | Purple 4621 | NA | CCS HAU | India | Hexaploid | 0.70 | 5.57 | 3.45 | 11.30 | 67.23 | 41.60 |
| 312 | PV 18 | NA | CCS HAU | India | Hexaploid | 0.78 | 5.42 | 3.36 | 12.10 | 67.30 | 30.60 |
| 313 | RAJ 1114 | NA | CCS HAU | India | Hexaploid | 0.83 | 5.26 | 3.26 | 10.90 | 70.50 | 27.50 |
| 314 | RAJ 1482 | NA | CCS HAU | India | Hexaploid | 0.93 | 5.85 | 3.63 | 10.87 | 71.07 | 31.10 |
| 315 | RAJ 1972 | NA | CCS HAU | India | Hexaploid | 0.88 | 5.09 | 3.16 | 13.50 | 70.70 | 28.10 |
| 316 | RAJ 2184 | NA | CCS HAU | India | Hexaploid | 0.92 | 5.74 | 3.56 | 13.80 | 70.67 | 25.80 |
| 317 | RAJ 3077 | NA | CCS HAU | India | Hexaploid | 0.97 | 5.84 | 3.62 | 10.50 | 73.07 | 36.90 |
| 318 | RAJ 3765 | NA | CCS HAU | India | Hexaploid | 0.97 | 5.62 | 3.48 | 11.13 | 71.70 | 45.40 |
| 319 | RAJ 3777 | NA | CCS HAU | India | Hexaploid | 0.84 | 5.56 | 3.45 | 14.53 | 68.30 | 27.90 |
| 320 | RAJ 4037 | NA | CCS HAU | India | Hexaploid | 0.75 | 5.60 | 3.47 | 14.10 | 68.20 | 27.00 |
| 321 | RAJ 821 | NA | CCS HAU | India | Hexaploid | 0.77 | 6.57 | 4.08 | 10.90 | 70.63 | 35.80 |
| 322 | RIDLEY | NA | CCS HAU | India | Hexaploid | 0.94 | 6.01 | 3.73 | 12.10 | 71.40 | 47.20 |
| 323 | RMR 1 | NA | CCS HAU | India | Hexaploid | 0.84 | 4.84 | 3.00 | 11.20 | 72.40 | 40.20 |
| 324 | RNB 1001 | NA | CCS HAU | India | Hexaploid | 0.79 | 6.55 | 4.06 | 9.43 | 73.50 | 39.20 |
| 325 | RS 3101 | NA | CCS HAU | India | Hexaploid | 0.79 | 5.67 | 3.52 | 10.40 | 74.13 | 32.00 |
| 326 | RW 3016 | NA | CCS HAU | India | Hexaploid | 0.68 | 4.67 | 2.89 | 11.30 | 72.40 | 33.50 |
| 327 | RW 346 | NA | CCS HAU | India | Hexaploid | 0.88 | 4.44 | 2.75 | 11.90 | 71.20 | 39.10 |
| 328 | RWP 2017-21 | NA | CCS HAU | India | Hexaploid | 0.79 | 4.91 | 3.04 | 11.63 | 70.77 | 34.20 |
| 329 | SAFED LERMA | NA | CCS HAU | India | Hexaploid | 0.85 | 5.41 | 3.35 | 11.90 | 71.13 | 27.40 |
| 330 | SAGARIKA | NA | CCS HAU | India | Hexaploid | 0.97 | 7.20 | 4.46 | 12.40 | 70.10 | 32.00 |
| 331 | SHARBATI SONORA | NA | CCS HAU | India | Hexaploid | 0.82 | 5.66 | 3.51 | 12.00 | 70.90 | 42.00 |
| 332 | SKW 196 | NA | CCS HAU | India | Hexaploid | 0.80 | 5.43 | 3.37 | 16.10 | 66.40 | 22.65 |
| 333 | SONALIKA | NA | CCS HAU | India | Hexaploid | 0.74 | 5.55 | 3.44 | 12.53 | 71.10 | 33.20 |
| 334 | SONORA 64 | NA | CCS HAU | India | Hexaploid | 0.86 | 5.27 | 3.27 | 12.50 | 69.70 | 22.70 |
| 335 | TAWA 267 | NA | CCS HAU | India | Hexaploid | 0.79 | 5.59 | 3.47 | 12.03 | 71.40 | 31.70 |
| 336 | Triticale 4 | NA | CCS HAU | India | Hexaploid | 0.56 | 6.30 | 3.91 | 9.90 | 71.43 | 45.70 |
| 337 | Triticale 5 | NA | CCS HAU | India | Hexaploid | 0.56 | 5.73 | 3.55 | 10.50 | 71.40 | 43.20 |
| 338 | Triticale 7 | NA | CCS HAU | India | Hexaploid | 0.79 | 4.94 | 3.06 | 11.60 | 70.17 | 46.90 |
| 339 | Triticale 8 | NA | CCS HAU | India | Hexaploid | 0.68 | 5.90 | 3.66 | 11.90 | 69.60 | 43.50 |
| 340 | UP 1109 | NA | CCS HAU | India | Hexaploid | 0.92 | 4.87 | 3.02 | 11.97 | 72.70 | 38.00 |
| 341 | UP 115 | NA | CCS HAU | India | Hexaploid | 0.96 | 6.01 | 3.73 | 12.13 | 70.90 | 42.10 |
| 342 | UP 2003 | NA | CCS HAU | India | Hexaploid | 0.83 | 5.49 | 3.40 | 12.57 | 70.60 | 30.50 |
| 343 | UP 2121 | NA | CCS HAU | India | Hexaploid | 0.82 | 5.35 | 3.32 | 13.03 | 69.80 | 33.40 |
| 344 | UP 215 | NA | CCS HAU | India | Hexaploid | 0.72 | 5.72 | 3.55 | 13.70 | 68.20 | 23.20 |
| 345 | UP 2338 | NA | CCS HAU | India | Hexaploid | 0.75 | 5.96 | 3.69 | 10.70 | 71.30 | 49.60 |
| 346 | UP 2425 | NA | CCS HAU | India | Hexaploid | 0.84 | 6.46 | 4.01 | 11.20 | 71.57 | 49.50 |
| 347 | UP 2473 | NA | CCS HAU | India | Hexaploid | 0.92 | 6.67 | 4.13 | 12.37 | 70.90 | 37.70 |
| 348 | UP 2565 | NA | CCS HAU | India | Hexaploid | 0.92 | 6.63 | 4.11 | 11.27 | 72.60 | 46.00 |
| 349 | UP 262 | NA | CCS HAU | India | Hexaploid | 0.79 | 5.38 | 3.33 | 11.50 | 72.20 | 36.90 |
| 350 | UP 2660 | NA | CCS HAU | India | Hexaploid | 0.50 | 6.46 | 4.01 | 11.73 | 71.00 | 51.20 |
| 351 | UP 2835 | NA | CCS HAU | India | Hexaploid | 0.73 | 6.73 | 4.17 | 11.87 | 70.40 | 49.50 |
| 352 | UP 2865 | NA | CCS HAU | India | Hexaploid | 0.81 | 6.52 | 4.04 | 12.90 | 69.43 | 46.64 |
| 353 | UP 2902 | NA | CCS HAU | India | Hexaploid | 0.89 | 6.17 | 3.83 | 11.50 | 70.40 | 36.00 |
| 354 | UP 2906 | NA | CCS HAU | India | Hexaploid | 0.86 | 6.05 | 3.75 | 13.20 | 68.60 | 48.30 |
| 355 | UP 2981 | NA | CCS HAU | India | Hexaploid | 0.83 | 6.72 | 4.17 | 11.20 | 72.10 | 46.40 |
| 356 | UP 3043 | NA | CCS HAU | India | Hexaploid | 0.90 | 7.98 | 4.95 | 11.00 | 71.70 | 47.90 |
| 357 | UP 368 | NA | CCS HAU | India | Hexaploid | 0.70 | 5.41 | 3.35 | 13.50 | 69.60 | 27.00 |
| 358 | UTKALIKA | NA | CCS HAU | India | Hexaploid | 0.76 | 4.45 | 2.76 | 10.70 | 72.80 | 36.40 |
| 359 | VINATA (N8223) | NA | CCS HAU | India | Hexaploid | 0.79 | 4.69 | 2.91 | 10.80 | 71.57 | 33.70 |
| 360 | VL 401 | NA | CCS HAU | India | Hexaploid | 0.70 | 5.02 | 3.11 | 14.40 | 67.27 | 33.40 |
| 361 | VL 404 | NA | CCS HAU | India | Hexaploid | 0.74 | 5.51 | 3.42 | 13.30 | 70.27 | 37.20 |
| 362 | VL 421 | NA | CCS HAU | India | Hexaploid | 0.82 | 5.62 | 3.48 | 16.10 | 65.40 | 36.10 |
| 363 | VL 616 | NA | CCS HAU | India | Hexaploid | 0.80 | 5.19 | 3.22 | 14.30 | 68.80 | 35.40 |
| 364 | VL 738 | NA | CCS HAU | India | Hexaploid | 0.81 | 5.82 | 3.61 | 11.23 | 70.80 | 38.20 |
| 365 | VL 802 | NA | CCS HAU | India | Hexaploid | 0.76 | 5.95 | 3.69 | 10.27 | 71.10 | 36.40 |
| 366 | VL 804 | NA | CCS HAU | India | Hexaploid | 0.96 | 5.56 | 3.45 | 9.70 | 72.20 | 34.70 |
| 367 | VL 829 | NA | CCS HAU | India | Hexaploid | 0.88 | 6.41 | 3.98 | 13.00 | 68.60 | 30.70 |
| 368 | VL 832 | NA | CCS HAU | India | Hexaploid | 0.78 | 5.75 | 3.57 | 10.40 | 72.20 | 41.80 |
| 369 | WB02 | NA | CCS HAU | India | Hexaploid | 0.53 | 5.84 | 3.62 | 12.90 | 70.20 | 41.60 |
| 370 | WG 357 | NA | CCS HAU | India | Hexaploid | 0.80 | 5.31 | 3.29 | 10.20 | 73.90 | 41.40 |
| 371 | WG 377 | NA | CCS HAU | India | Hexaploid | 0.79 | 5.55 | 3.44 | 10.20 | 72.80 | 40.10 |
| 372 | WH 1021 | NA | CCS HAU | India | Hexaploid | 1.12 | 5.71 | 3.54 | 14.67 | 66.17 | 36.64 |
| 373 | WH 1025 | NA | CCS HAU | India | Hexaploid | 0.90 | 4.79 | 2.97 | 12.90 | 70.60 | 48.70 |
| 374 | WH 1061 | NA | CCS HAU | India | Hexaploid | 0.93 | 5.98 | 3.71 | 12.87 | 70.00 | 42.34 |
| 375 | WH 1062 | NA | CCS HAU | India | Hexaploid | 0.86 | 6.33 | 3.93 | 13.10 | 69.10 | 44.54 |
| 376 | WH 1063 | NA | CCS HAU | India | Hexaploid | 0.96 | 5.93 | 3.68 | 12.37 | 69.30 | 36.20 |
| 377 | WH 1080 | NA | CCS HAU | India | Hexaploid | 0.97 | 5.48 | 3.40 | 12.83 | 68.80 | 41.44 |
| 378 | WH 1105 | NA | CCS HAU | India | Hexaploid | 1.10 | 6.28 | 3.89 | 14.03 | 67.80 | 43.60 |
| 379 | WH 1123 | NA | CCS HAU | India | Hexaploid | 0.91 | 4.79 | 2.97 | 13.17 | 69.53 | 45.94 |
| 380 | WH 1124 | NA | CCS HAU | India | Hexaploid | 0.90 | 5.71 | 3.54 | 12.10 | 70.00 | 41.24 |
| 381 | WH 1126 | NA | CCS HAU | India | Hexaploid | 0.78 | 6.16 | 3.82 | 12.40 | 69.17 | 47.84 |
| 382 | WH 1127 | NA | CCS HAU | India | Hexaploid | 0.94 | 5.02 | 3.11 | 11.50 | 70.73 | 48.60 |
| 383 | WH 1129 | NA | CCS HAU | India | Hexaploid | 1.04 | 6.05 | 3.75 | 14.37 | 67.40 | 42.00 |
| 384 | WH 1131 | NA | CCS HAU | India | Hexaploid | 0.93 | 6.39 | 3.96 | 13.77 | 66.80 | 46.60 |
| 385 | WH 1132 | NA | CCS HAU | India | Hexaploid | 0.93 | 6.96 | 4.32 | 13.90 | 67.90 | 38.54 |
| 386 | WH 1133 | NA | CCS HAU | India | Hexaploid | 0.93 | 6.16 | 3.82 | 12.30 | 69.10 | 36.74 |
| 387 | WH 1134 | NA | CCS HAU | India | Hexaploid | 0.82 | 5.71 | 3.54 | 14.00 | 67.53 | 42.64 |
| 388 | WH 1135 | NA | CCS HAU | India | Hexaploid | 1.09 | 4.91 | 3.04 | 13.00 | 68.80 | 39.94 |
| 389 | WH 1136 | NA | CCS HAU | India | Hexaploid | 1.00 | 4.68 | 2.90 | 13.07 | 70.50 | 44.24 |
| 390 | WH 1137 | NA | CCS HAU | India | Hexaploid | 0.95 | 6.73 | 4.17 | 15.10 | 66.23 | 45.70 |
| 391 | WH 1138 | NA | CCS HAU | India | Hexaploid | 0.86 | 4.11 | 2.55 | 13.80 | 68.67 | 35.44 |
| 392 | WH 1140 | NA | CCS HAU | India | Hexaploid | 0.96 | 5.71 | 3.54 | 13.97 | 67.27 | 44.64 |
| 393 | WH 1142 | NA | CCS HAU | India | Hexaploid | 0.80 | 4.91 | 3.04 | 13.03 | 68.93 | 38.74 |
| 394 | WH 1151 | NA | CCS HAU | India | Hexaploid | 0.97 | 5.48 | 3.40 | 12.70 | 69.20 | 49.10 |
| 395 | WH 1152 | NA | CCS HAU | India | Hexaploid | 1.07 | 5.93 | 3.68 | 12.27 | 69.07 | 49.50 |
| 396 | WH 1153 | NA | CCS HAU | India | Hexaploid | 1.03 | 6.16 | 3.82 | 13.50 | 68.13 | 44.24 |
| 397 | WH 1156 | NA | CCS HAU | India | Hexaploid | 1.14 | 6.05 | 3.75 | 11.57 | 69.67 | 42.60 |
| 398 | WH 1157 | NA | CCS HAU | India | Hexaploid | 0.80 | 4.79 | 2.97 | 13.50 | 69.43 | 45.00 |
| 399 | WH 1159 | NA | CCS HAU | India | Hexaploid | 0.97 | 5.25 | 3.25 | 14.77 | 66.00 | 38.40 |
| 400 | WH 1160 | NA | CCS HAU | India | Hexaploid | 1.31 | 5.82 | 3.61 | 14.20 | 67.70 | 41.40 |
| 401 | WH 1164 | NA | CCS HAU | India | Hexaploid | 0.87 | 5.25 | 3.25 | 12.13 | 70.20 | 35.14 |
| 402 | WH 1165 | NA | CCS HAU | India | Hexaploid | 0.97 | 6.51 | 4.03 | 13.60 | 68.50 | 45.84 |
| 403 | WH 1175 | NA | CCS HAU | India | Hexaploid | 1.04 | 4.91 | 3.04 | 13.33 | 69.93 | 44.20 |
| 404 | WH 1182 | NA | CCS HAU | India | Hexaploid | 1.06 | 5.59 | 3.47 | 13.90 | 68.70 | 36.90 |
| 405 | WH 1184 | NA | CCS HAU | India | Hexaploid | 0.96 | 6.28 | 3.89 | 13.73 | 68.20 | 40.12 |
| 406 | WH 1186 | NA | CCS HAU | India | Hexaploid | 0.91 | 5.14 | 3.18 | 12.40 | 69.47 | 48.30 |
| 407 | WH 1188 | NA | CCS HAU | India | Hexaploid | 0.92 | 5.59 | 3.47 | 13.20 | 69.50 | 38.64 |
| 408 | WH 1202 | NA | CCS HAU | India | Hexaploid | 0.99 | 6.28 | 3.89 | 13.10 | 69.30 | 44.30 |
| 409 | WH 1218 | NA | CCS HAU | India | Hexaploid | 1.16 | 5.02 | 3.11 | 13.50 | 68.20 | 42.70 |
| 410 | WH 1235 | NA | CCS HAU | India | Hexaploid | 0.84 | 4.34 | 2.69 | 13.00 | 67.70 | 45.40 |
| 411 | WH 1270 | NA | CCS HAU | India | Hexaploid | 0.85 | 4.22 | 2.62 | 13.50 | 68.60 | 47.24 |
| 412 | WH 147 | NA | CCS HAU | India | Hexaploid | 0.96 | 6.30 | 3.91 | 14.03 | 69.03 | 36.40 |
| 413 | WH 157 | NA | CCS HAU | India | Hexaploid | 0.95 | 5.02 | 3.11 | 13.63 | 70.47 | 45.80 |
| 414 | WH 283 | NA | CCS HAU | India | Hexaploid | 1.11 | 4.68 | 2.90 | 13.40 | 69.20 | 43.54 |
| 415 | WH 522 | NA | CCS HAU | India | Hexaploid | 0.97 | 5.02 | 3.11 | 12.90 | 68.30 | 34.20 |
| 416 | WH 542 | NA | CCS HAU | India | Hexaploid | 1.00 | 7.42 | 4.60 | 14.40 | 66.67 | 36.80 |
| 417 | WH 711 | NA | CCS HAU | India | Hexaploid | 0.87 | 5.25 | 3.25 | 13.53 | 69.50 | 45.10 |
| 418 | WH 715 | NA | CCS HAU | India | Hexaploid | 0.97 | 5.59 | 3.47 | 13.87 | 66.73 | 35.04 |
| 419 | WH 787 | NA | CCS HAU | India | Hexaploid | 0.98 | 6.28 | 3.89 | 13.67 | 68.10 | 42.94 |
| 420 | WH 789 | NA | CCS HAU | India | Hexaploid | 0.97 | 5.85 | 3.63 | 14.47 | 67.77 | 46.20 |
| 421 | WHD 943 | NA | CCS HAU | India | Tetraploid | 1.05 | 4.04 | 2.50 | 12.70 | 69.97 | 44.20 |
| 422 | WL 1562 | NA | CCS HAU | India | Hexaploid | 0.89 | 5.18 | 3.21 | 13.70 | 70.27 | 36.50 |
| 423 | WL 2265 | NA | CCS HAU | India | Hexaploid | 0.84 | 4.92 | 3.05 | 10.67 | 72.50 | 41.90 |
| 424 | WL 410 | NA | CCS HAU | India | Hexaploid | 1.04 | 6.32 | 3.92 | 13.40 | 68.10 | 33.10 |
| 425 | WL 711 | NA | CCS HAU | India | Hexaploid | 0.92 | 5.73 | 3.55 | 11.20 | 72.10 | 28.60 |
| 426 | WW 360 | NA | CCS HAU | India | Hexaploid | 0.76 | 6.54 | 4.05 | 10.10 | 72.10 | 48.30 |
| 427 | Aconchi-89 (Triticum durum) | Aconchi-89 | NBPGR | India | Tetraploid | 0.45 | 4.85 | 3.01 | 19.00 | 75.60 | 59.01 |
| 428 | PDW-233 (Triticum durum) | PDW-233 | NBPGR | India | Tetraploid | 0.30 | 4.26 | 2.64 | 17.87 | 72.83 | 63.20 |
| 429 | PDW-274 (Triticum durum) | PDW-274 | NBPGR | India | Tetraploid | 0.37 | 5.30 | 3.28 | 16.30 | 85.33 | 57.87 |
| 430 | PDW-291 (Triticum durum) | PDW-291 | NBPGR | India | Tetraploid | 0.39 | 5.30 | 3.28 | 19.73 | 72.80 | 60.27 |
| 431 | PDW-314 (Triticum durum) | PDW-314 | NBPGR | India | Tetraploid | 0.41 | 5.11 | 3.17 | 17.43 | 84.80 | 57.83 |
| 432 | Syn 11 | NA | CCS HAU | India | Hexaploid | 0.86 | 5.83 | 3.62 | 10.50 | 69.90 | 48.20 |
| 433 | Syn 150 | NA | CCS HAU | India | Hexaploid | 0.77 | 5.79 | 3.59 | 11.40 | 71.97 | 49.70 |
| 434 | Syn 2 | NA | CCS HAU | India | Hexaploid | 0.79 | 6.43 | 3.98 | 11.87 | 72.30 | 44.20 |
| 435 | Syn 22 | NA | CCS HAU | India | Hexaploid | 0.72 | 5.91 | 3.67 | 12.50 | 67.73 | 39.00 |
| 436 | Syn 24 | NA | CCS HAU | India | Hexaploid | 0.79 | 6.33 | 3.93 | 10.70 | 69.40 | 43.80 |
| 437 | Syn 28 | NA | CCS HAU | India | Hexaploid | 0.76 | 6.61 | 4.10 | 12.50 | 68.23 | 49.10 |
| 438 | Syn 4 | NA | CCS HAU | India | Hexaploid | 0.75 | 6.59 | 4.08 | 13.10 | 67.67 | 38.10 |
| 439 | Syn 40 | NA | CCS HAU | India | Hexaploid | 0.73 | 5.92 | 3.67 | 12.00 | 68.30 | 45.20 |
| 440 | Syn 5 | NA | CCS HAU | India | Hexaploid | 0.69 | 6.94 | 4.30 | 11.03 | 69.13 | 47.40 |
| 441 | Syn 7 | NA | CCS HAU | India | Hexaploid | 0.79 | 5.17 | 3.21 | 11.60 | 71.43 | 38.50 |
| 442 | *Aegilops dicoccoides-4640* | W- 4640 | NBPGR | India | Wild | 0.36 | 4.61 | 2.86 | 15.80 | 43.17 | 17.54 |
| 443 | *Aegilops dicoccoides-744* | W- 744 | NBPGR | India | Wild | 0.47 | 5.77 | 3.58 | 17.40 | 47.27 | 20.74 |
| 444 | *Aegilops dicoccoides-794* | W- 794 | NBPGR | India | Wild | 0.32 | 5.23 | 3.24 | 15.57 | 61.03 | 23.58 |
| 445 | *Aegilops kotschyi-600* | W- 600 | NBPGR | India | Wild | 2.02 | 7.73 | 4.79 | 18.97 | 48.47 | 8.23 |
| 446 | *Aegilops kotschyi-601* | W- 601 | NBPGR | India | Wild | 1.67 | 10.34 | 6.41 | 20.27 | 52.10 | 8.45 |
| 447 | *Aegilops kotschyi-396* | W- 396 | NBPGR | India | Wild | 2.00 | 9.50 | 5.89 | 19.83 | 49.13 | 8.82 |
| 448 | *Aegilops kotschyi-387* | W- 387 | NBPGR | India | Wild | 2.74 | 6.95 | 4.31 | 18.27 | 55.87 | 8.34 |
| 449 | *Aegilops kotschyi-390* | W- 390 | NBPGR | India | Wild | 2.54 | 8.19 | 5.08 | 23.33 | 53.33 | 9.27 |
| 450 | *Aegilops kotschyi-391* | W- 391 | NBPGR | India | Wild | 3.00 | 8.19 | 5.08 | 23.07 | 52.20 | 10.93 |
| 451 | *Aegilops kotschyi-394* | W- 394 | NBPGR | India | Wild | 2.27 | 7.35 | 4.56 | 18.43 | 63.27 | 6.86 |
| 452 | *Aegilops kotschyi-3790* | W- 3790 | NBPGR | India | Wild | 2.40 | 7.26 | 4.50 | 22.80 | 49.77 | 9.07 |
| 453 | *Triticum monococcum-463* | W- 463 | NBPGR | India | Wild | 0.50 | 4.46 | 2.77 | 19.77 | 58.90 | 20.39 |
| 454 | *Triticum monococcum-482* | W- 482 | NBPGR | India | Wild | 0.58 | 4.62 | 2.84 | 19.23 | 55.57 | 24.99 |
| 455 | *Triticum monococcum-487* | W- 487 | NBPGR | India | Wild | 0.58 | 3.91 | 2.43 | 17.47 | 53.03 | 24.91 |
| 456 | *Triticum monococcum-14087* | W- 14087 | NBPGR | India | Wild | 0.72 | 5.24 | 3.25 | 19.57 | 46.10 | 20.79 |
| 457 | *Aegilops peregrina-3477* | W- 3477 | NBPGR | India | Wild | 3.38 | 8.79 | 5.45 | 22.43 | 31.67 | 8.01 |
| 458 | *Aegilops peregrina-3519* | W- 3519 | NBPGR | India | Wild | 2.91 | 8.90 | 5.52 | 22.50 | 35.87 | 18.05 |
| 459 | *Aegilops peregrina-3791* | W- 3791 | NBPGR | India | Wild | 3.13 | 7.57 | 4.69 | 23.60 | 26.27 | 10.12 |
| 460 | *Aegilops peregrina-13772* | W- 13772 | NBPGR | India | Wild | 3.19 | 7.50 | 4.65 | 24.27 | 48.00 | 11.71 |
| 461 | *Aegilops peregrina-3772* | W- 3772 | NBPGR | India | Wild | 3.18 | 6.59 | 4.08 | 19.37 | 47.13 | 13.24 |
| 462 | *Aegilops peregrina-631* | W- 631 | NBPGR | India | Wild | 3.65 | 7.25 | 4.49 | 21.37 | 27.93 | 7.62 |
| 463 | *Aegilops peregrina-629* | W- 629 | NBPGR | India | Wild | 3.73 | 9.04 | 5.60 | 22.30 | 25.17 | 11.49 |
| 464 | *Aegilops speltoides-3581* | W- 3581 | NBPGR | India | Wild | 1.25 | 6.83 | 4.69 | 19.03 | 55.63 | 6.62 |
| 465 | *Aegilops speltoides-3804* | W- 3804 | NBPGR | India | Wild | 0.97 | 5.63 | 3.49 | 19.20 | 55.00 | 9.74 |
| 466 | *Aegilops speltoides-3808* | W- 3808 | NBPGR | India | Wild | 0.93 | 5.57 | 3.43 | 19.37 | 55.63 | 8.53 |
| 467 | *Aegilops tauschii-4* | W- 4 | NBPGR | India | Wild | 2.69 | 8.05 | 4.99 | 21.93 | 57.47 | 7.12 |
| 468 | *Aegilops tauschii-108* | W- 108 | NBPGR | India | Wild | 2.89 | 6.90 | 4.28 | 16.20 | 63.57 | 7.21 |
| 469 | *Aegilops tauschii-199* | W- 109 | NBPGR | India | Wild | 2.98 | 7.81 | 4.84 | 19.67 | 65.83 | 12.05 |
| 470 | *Aegilops tauschii-265* | W- 265 | NBPGR | India | Wild | 3.03 | 6.78 | 4.20 | 18.50 | 71.20 | 10.94 |
| 471 | *Aegilops tauschii-282* | W- 282 | NBPGR | India | Wild | 2.93 | 7.51 | 4.66 | 18.87 | 76.37 | 13.31 |
| 472 | *Aegilops tauschii-14232* | W- 14232 | NBPGR | India | Wild | 2.26 | 7.08 | 4.39 | 20.10 | 63.47 | 8.25 |
| 473 | *Aegilops tauschii-14096* | W- 14096 | NBPGR | India | Wild | 2.79 | 6.63 | 4.11 | 21.50 | 61.27 | 10.45 |
| 474 | *Aegilops tauschii-153* | W- 153 | NBPGR | India | Wild | 3.08 | 8.89 | 5.51 | 22.90 | 57.03 | 10.88 |
| 475 | *Aegilops umbellulata-517* | W- 517 | NBPGR | India | Wild | 2.51 | 7.14 | 4.43 | 20.27 | 67.67 | 14.86 |
| 476 | *Aegilops umbellulata-520* | W- 520 | NBPGR | India | Wild | 2.47 | 5.65 | 3.50 | 17.33 | 67.00 | 15.91 |
| 477 | *Aegilops araraticum-4761* | W- 4761 | NBPGR | India | Wild | 0.49 | 4.23 | 2.63 | 18.77 | 65.67 | 24.64 |
| 478 | *Aegilops araraticum-4692* | W- 4692 | NBPGR | India | Wild | 1.07 | 8.89 | 5.51 | 21.90 | 65.10 | 34.56 |
|  | **Mean** |  |  |  |  | **0.93** | **5.77** | **3.58** | **13.37** | **68.51** | **36.74** |

**Table S2.** List of 50 best genotypes selected from a panel of 478 wheat genotypes for **dietary fibre components (β-glucan and arabinoxylan) alongside other grain composition traits including protein, starch, and thousand-grain weight**.

| **Sr.** | **β-glucan (% dry wt.)** | **Genotypes** | **Arabinoxylan (% dry wt.)** | **Genotypes** | **D-Xylose (% dry wt.)** | **Genotypes** | **Protein (% dry wt.)** | **Genotypes** | **Starch (% dry wt.)** | **Genotypes** | **Thousand-Grain Weight (g)** | **Genotypes** |
| --- | --- | --- | --- | --- | --- | --- | --- | --- | --- | --- | --- | --- |
| **1** | 3.73 | *Ae.peregrina*-629 | 10.34 | *Ae.kotschyi* -601 | 6.41 | *Ae.kotschyi* -601 | 24.27 | *Ae.peregrina*-13772 | 85.33 | PDW-274 | 63.20 | PDW-233 |
| **2** | 3.65 | *Ae.peregrina*-631 | 9.50 | *Ae.kotschyi*-396 | 5.89 | *Ae.kotschyi*-396 | 23.60 | *Ae.peregrina*-3791 | 84.80 | PDW-314 | 60.27 | PDW-291 |
| **3** | 3.38 | *Ae.peregrina*-3477 | 9.04 | *Ae.peregrina*-629 | 5.60 | *Ae.peregrina*-629 | 23.33 | *Ae.kotschyi*-390 | 76.37 | *Ae.tauschii* -282 | 59.01 | Aconchi-89 |
| **4** | 3.19 | *Ae.peregrina*-13772 | 8.90 | *Ae.peregrina*-3519 | 5.52 | *Ae.peregrina*-3519 | 23.07 | *Ae.kotschyi*-391 | 75.60 | Aconchi-89 | 57.87 | PDW-274 |
| **5** | 3.18 | *Ae.peregrina*-3772 | 8.89 | *Ae.tauschii* -153 | 5.51 | *Ae.tauschii* -153 | 22.90 | *Ae.tauschii* -153 | 74.13 | RS 3101 | 57.83 | PDW-314 |
| **6** | 3.13 | *Ae.peregrina*-3791 | 8.89 | *Ae.araraticum*-4692 | 5.51 | *Ae.araraticum*-4692 | 22.80 | *Ae.kotschyi*-3790 | 73.90 | WG 357 | 53.80 | PBW 706 |
| **7** | 3.08 | *Ae.tauschii* -153 | 8.79 | *Ae.peregrina*-3477 | 5.45 | *Ae.peregrina*-3477 | 22.50 | *Ae.peregrina*-3519 | 73.80 | DBW 11 | 53.20 | NW 2306 |
| **8** | 3.03 | *Ae.tauschii* -265 | 8.19 | *Ae.kotschyi*-390 | 5.08 | *Ae.kotschyi*-390 | 22.43 | *Ae.peregrina*-3477 | 73.80 | PBW 120 | 52.44 | PBW 165 |
| **9** | 3.00 | *Ae.kotschyi*-391 | 8.19 | *Ae.kotschyi*-391 | 5.08 | *Ae.kotschyi*-391 | 22.30 | *Ae.peregrina*-629 | 73.73 | HS 1138-6-4 (SHAILJA) | 52.44 | PBW 726 |
| **10** | 2.98 | *Ae.tauschii* -199 | 8.05 | *Ae.tauschii* -4 | 4.99 | *Ae.tauschii* -4 | 21.93 | *Ae.tauschii* -4 | 73.70 | PBW 138 | 52.30 | NW 707 |
| **11** | 2.93 | *Ae.tauschii* -282 | 7.98 | UP 3043 | 4.95 | UP 3043 | 21.90 | *Ae.araraticum*-4692 | 73.50 | RNB 1001 | 52.10 | P 11638 |
| **12** | 2.91 | *Ae.peregrina*-3519 | 7.95 | PBW 475 | 4.93 | PBW 475 | 21.50 | *Ae.tauschii*-14096 | 73.40 | HD 2380 | 51.70 | DBW 17 |
| **13** | 2.89 | *Ae.tauschii* -108 | 7.81 | *Ae.tauschii* -199 | 4.84 | *Ae.tauschii* -199 | 21.37 | *Ae.peregrina*-631 | 73.30 | PBW 154 | 51.60 | PBW 796 |
| **14** | 2.79 | *Ae.tauschii*-14096 | 7.73 | *Ae.kotschyi* -600 | 4.79 | *Ae.kotschyi* -600 | 20.27 | *Ae.kotschyi* -601 | 73.20 | HD 2327 | 51.50 | K 4117 |
| **15** | 2.74 | *Ae.kotschyi*-387 | 7.61 | C 306 | 4.72 | C 306 | 20.27 | *Ae.umbellulata* -517 | 73.10 | MONDHYA 3?2 | 51.20 | UP 2660 |
| **16** | 2.69 | *Ae.tauschii* -4 | 7.57 | *Ae.peregrina*-3791 | 4.69 | *Ae.peregrina*-3791 | 20.10 | *Ae.tauschii*-14232 | 73.07 | RAJ 3077 | 51.14 | PBW 123 |
| **17** | 2.54 | *Ae.kotschyi*-390 | 7.54 | PBW 175 | 4.69 | *Ae.speltoides*-3581 | 19.83 | *Ae.kotschyi*-396 | 73.00 | BRW 3806 | 50.90 | HD 2968 |
| **18** | 2.51 | *Ae.umbellulata* -517 | 7.51 | *Ae.tauschii* -282 | 4.68 | PBW 175 | 19.77 | *T.monococcum*-463 | 72.90 | HUW 234 | 50.50 | PBW 621 |
| **19** | 2.47 | *Ae.umbellulata*-520 | 7.50 | *Ae.peregrina*-13772 | 4.66 | *Ae.tauschii* -282 | 19.73 | PDW-291 | 72.90 | KRL 1-4 | 50.50 | PBW 709 |
| **20** | 2.40 | *Ae.kotschyi*-3790 | 7.49 | HD 3219 | 4.65 | *Ae.peregrina*-13772 | 19.67 | *Ae.tauschii* -199 | 72.83 | PDW-233 | 50.20 | PBW 752 |
| **21** | 2.27 | *Ae.kotschyi*-394 | 7.45 | PBW 533 | 4.64 | HD 3219 | 19.57 | *T.monococcum*-14087 | 72.80 | K 65 | 50.00 | PBW 560 |
| **22** | 2.26 | *Ae.tauschii*-14232 | 7.42 | WH 542 | 4.62 | PBW 533 | 19.37 | *Ae.peregrina*-3772 | 72.80 | UTKALIKA | 49.80 | NP 710 |
| **23** | 2.02 | *Ae.kotschyi* -600 | 7.35 | PBW 503 | 4.60 | WH 542 | 19.37 | *Ae.speltoides*-3808 | 72.80 | WG 377 | 49.80 | PMW 621-50 |
| **24** | 2.00 | *Ae.kotschyi*-396 | 7.35 | *Ae.kotschyi*-394 | 4.56 | PBW 503 | 19.23 | *T.monococcum*-482 | 72.80 | PDW-291 | 49.70 | HD 3182 |
| **25** | 1.67 | *Ae.kotschyi* -601 | 7.34 | HD 2987 | 4.56 | *Ae.kotschyi*-394 | 19.20 | *Ae.speltoides*-3804 | 72.70 | NP 100 | 49.70 | K 65 |
| **26** | 1.31 | WH 1160 | 7.32 | DBW 90 | 4.55 | HD 2987 | 19.03 | *Ae.speltoides*-3581 | 72.70 | UP 1109 | 49.70 | Syn 150 |
| **27** | 1.25 | *Ae.speltoides*-3581 | 7.26 | *Ae.kotschyi*-3790 | 4.54 | DBW 90 | 19.00 | Aconchi-89 | 72.60 | NP 745 | 49.60 | NBRL 2015-07 |
| **28** | 1.20 | GW 10 | 7.25 | *Ae.peregrina*-631 | 4.50 | *Ae.kotschyi*-3790 | 18.97 | *Ae.kotschyi* -600 | 72.60 | UP 2565 | 49.60 | PBW 138 |
| **29** | 1.17 | K 9644 (ATAL) | 7.24 | HD 2851 | 4.49 | HD 2851 | 18.87 | AJANTA | 72.57 | HS 86 | 49.60 | UP 2338 |
| **30** | 1.16 | PBW 766 | 7.20 | SAGARIKA | 4.49 | *Ae.peregrina*-631 | 18.87 | *Ae.tauschii* -282 | 72.57 | KRL 19 | 49.50 | HI 617 (SUJATA) |
| **31** | 1.16 | WH 1218 | 7.14 | *Ae.umbellulata* -517 | 4.46 | SAGARIKA | 18.77 | *Ae.araraticum*-4761 | 72.50 | HD 2278 (PARVATI) | 49.50 | UP 2425 |
| **32** | 1.14 | PBW 825 | 7.10 | HD 2967 | 4.43 | *Ae.umbellulata* -517 | 18.50 | *Ae.tauschii* -265 | 72.50 | WL 2265 | 49.50 | UP 2835 |
| **33** | 1.14 | WH 1156 | 7.08 | *Ae.tauschii*-14232 | 4.40 | HD 2967 | 18.43 | *Ae.kotschyi*-394 | 72.43 | KSML 3 | 49.50 | WH 1152 |
| **34** | 1.13 | DBW 95 | 7.04 | HD 3086 | 4.39 | *Ae.tauschii*-14232 | 18.27 | *Ae.kotschyi*-387 | 72.43 | NP 824 | 49.40 | PBW 757 |
| **35** | 1.12 | WH 1021 | 6.96 | WH 1132 | 4.37 | HD 3086 | 17.90 | DL 784-3 (VAISHALI) | 72.40 | HD 2385 | 49.20 | DBW 16 |
| **36** | 1.11 | PBW 175 | 6.95 | *Ae.kotschyi*-387 | 4.32 | WH 1132 | 17.87 | PDW-233 | 72.40 | NP 761 | 49.10 | HUW 540 |
| **37** | 1.11 | WH 283 | 6.94 | HD 3059 | 4.31 | *Ae.kotschyi*-387 | 17.50 | CPAN 3004 (SANGAM) | 72.40 | RMR 1 | 49.10 | WH 1151 |
| **38** | 1.10 | DBW 222 | 6.94 | Syn 5 | 4.30 | HD 3059 | 17.47 | *T.monococcum*-487 | 72.40 | RW 3016 | 49.10 | Syn 28 |
| **39** | 1.10 | PBW 163 | 6.93 | PBW 766 | 4.30 | PBW 766 | 17.43 | PDW-314 | 72.30 | HD 2270 | 48.70 | C 591 |
| **40** | 1.10 | WH 1105 | 6.90 | *Ae.tauschii* -108 | 4.30 | Syn 5 | 17.40 | *Ae. dicoccoides*-744 | 72.30 | Syn 2 | 48.70 | DBW 233 |
| **41** | 1.09 | WH 1135 | 6.87 | DBW 136 | 4.28 | *Ae.tauschii* -108 | 17.33 | *Ae.umbellulata*-520 | 72.23 | HP 1744 (RAJESHWARI) | 48.70 | PBW 769 |
| **42** | 1.07 | HW 2045 (KAUSHAMBI) | 6.86 | HUW 540 | 4.26 | DBW 136 | 17.30 | CHHOTI LERMA | 72.20 | HD 3182 | 48.70 | WH 1025 |
| **43** | 1.07 | PBW 396 | 6.85 | HI 617 (SUJATA) | 4.25 | HI 617 (SUJATA) | 17.20 | CPAN 1796 | 72.20 | HS 295 | 48.60 | WH 1127 |
| **44** | 1.07 | PBW 769 | 6.85 | PBW 163 | 4.25 | HUW 540 | 17.20 | NP 12 | 72.20 | UP 262 | 48.40 | NW 7041 |
| **45** | 1.07 | WH 1152 | 6.84 | HI 1500 (AMRITA) | 4.25 | PBW 163 | 17.10 | DWR 195 (ANURADHA) | 72.20 | VL 804 | 48.30 | PBW 502 |
| **46** | 1.07 | *Ae.araraticum*-4692 | 6.84 | P 11638 | 4.24 | HI 1500 (AMRITA) | 16.97 | C 591 | 72.20 | VL 832 | 48.30 | UP 2906 |
| **47** | 1.06 | HPBW 07 | 6.83 | *Ae.speltoides*-3581 | 4.24 | P 11638 | 16.90 | GW 10 | 72.10 | DBW 129 | 48.30 | WH 1186 |
| **48** | 1.06 | PBW 533 | 6.79 | BRW 7342 | 4.21 | BRW 7342 | 16.80 | DWR 16 (KEERTHI) | 72.10 | HD 2402 | 48.30 | WW 360 |
| **49** | 1.06 | WH 1182 | 6.78 | *Ae.tauschii* -265 | 4.20 | *Ae.tauschii* -265 | 16.43 | C 518 | 72.10 | K 9533 (NAINA) | 48.20 | DBW 14 |
| **50** | 1.05 | HD 2402 | 6.73 | HD 3011 | 4.17 | HD 3011 | 16.30 | DL 788-2 (VIDISHA) | 72.10 | PBW 54 | 48.20 | LOK 54 |


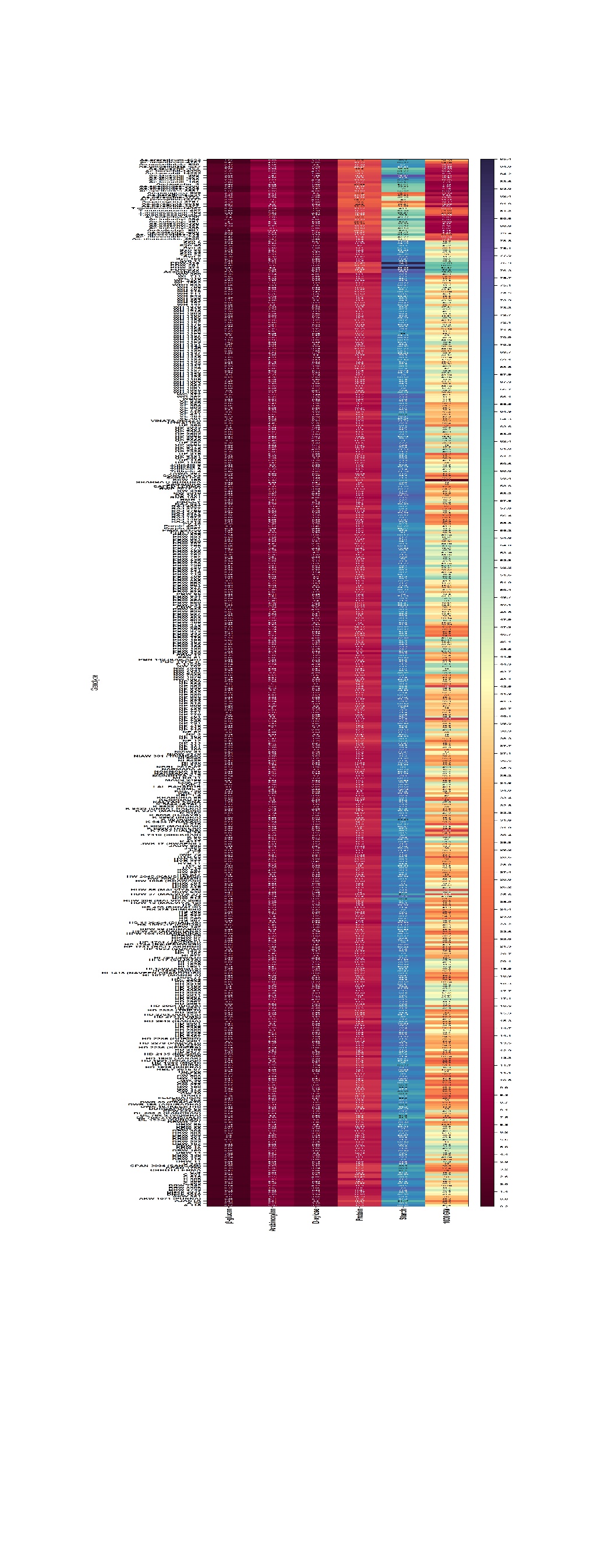


**Figure S1.** Heat map showing variation in wheat genotypes for their **dietary fibre components (β-glucan and arabinoxylan) alongside other grain composition traits including protein, starch, and thousand-grain weight**. Rows represent 478 wheat genotypes and columns the difference between the individual genotype and their variable parameters (β-glucan, arabinoxylan, D-xylose, protein, starch and thousand-grain weight). Colour of each cell indicates the value and magnitude of the main variable in the corresponding cell range.
